# Hematoma Resolution In Vivo Is Directed by Activating Transcription Factor 1

**DOI:** 10.1161/CIRCRESAHA.119.315528

**Published:** 2020-07-02

**Authors:** Anusha Seneviratne, Yumeng Han, Eunice Wong, Edward R.H. Walter, Lijun Jiang, Luke Cave, Nicholas J. Long, David Carling, Justin C. Mason, Dorian O. Haskard, Joseph J. Boyle

**Affiliations:** 1 From the National Heart and Lung Institute (A.S., Y.H., E.W., E.R.H.W., L.C., J.C.M., D.O.H., J.J.B.), Imperial College London Hammersmith Campus; 2 MRC London Institute of Medical Sciences (D.C.), Imperial College London Hammersmith Campus; 3 Molecular Sciences Research Hub, Imperial College London White City Campus (Y.H., E.W., E.R.H.W., L.J., N.J.L.).

**Keywords:** hemorrhage, inflammation, lipids, macrophages, oxidative stress

## Abstract

The efficient resolution of tissue hemorrhage is an important homeostatic function. In human macrophages in vitro, heme activates an AMPK (AMP-activated protein kinase)/ATF1 (activating transcription factor-1) pathway that directs Mhem macrophages through coregulation of HO-1 (heme oxygenase-1; *HMOX1*) and lipid homeostasis genes.

**Objective:**

We asked whether this pathway had an in vivo role in mice.

**Methods and Results:**

Perifemoral hematomas were used as a model of hematoma resolution. In mouse bone marrow–derived macrophages, heme induced HO-1, lipid regulatory genes including LXR (lipid X receptor), the growth factor IGF1 (insulin-like growth factor-1), and the splenic red pulp macrophage gene *Spic*. This response was lost in bone marrow–derived macrophages from mice deficient in AMPK (*Prkab1*^−/^^−^) or ATF1 (*Atf1*^−/−^). In vivo, femoral hematomas resolved completely between days 8 and 9 in littermate control mice (n=12), but were still present at day 9 in mice deficient in either AMPK (*Prkab1*^−/−^) or ATF1 (*Atf1*^−/−^; n=6 each). Residual hematomas were accompanied by increased macrophage infiltration, inflammatory activation and oxidative stress. We also found that fluorescent lipids and a fluorescent iron-analog were trafficked to lipid-laden and iron-laden macrophages respectively. Moreover erythrocyte iron and lipid abnormally colocalized in the same macrophages in *Atf1*^−/−^ mice. Therefore, iron-lipid separation was *Atf1*-dependent.

**Conclusions:**

Taken together, these data demonstrate that both AMPK and ATF1 are required for normal hematoma resolution.

## Abstract 2

Supplemental Digital Content is available in the text.

**Meet the First Author, see p 854**

The efficient resolution of tissue hemorrhage (and hematoma) is important in life-threatening human diseases, such as intracranial hemorrhage and intraplaque hemorrhage of atherosclerotic lesions.^1^ Hematomas in other sites, including skin, subcutaneous compartments, and musculoskeletal injury, are also common and important in trauma.^2,3^ The recurrent hematomas in hereditary hemophilia cause severe disability via the tissue reaction they elicit.^4–7^ Therefore, hematoma resolution is likely to be an important homeostatic and disease-preventing process in clinical and veterinary medicine.^3^ When successful, this process recycles erythrocyte components while minimizing inflammatory and oxidative stress from tissue hemoglobin.

The molecular understanding of the normal clearance of tissue hemorrhage is patchy.^1^ It is most studied in the context of intracerebral hemorrhage. Hemorrhage resolution is understood to be dependent on macrophages^8,9^ and promoted by agonists of PPARγ (peroxisome proliferator-activated nuclear receptor-gamma).^10^

One of the key effector enzymes in hemorrhage resolution is HO-1 (heme oxygenase-1).^11–14^ HO-1 is encoded by the gene *HMOX1* and has a very highly characterized enzyme function. Iron protoporphyrin-IX (heme) is the main prosthetic moiety of hemoglobin. HO-1 has a pocket that accepts heme, opens (decyclizes) the porphyrin ring, and removes the ferrous ion.^11–14^ This ultimately results in safe chelation of iron in ferritin and in generation of bilirubin.^12–14^ Classical bruise evolution (blue/black-brown-green-yellow) reflects HO-1 activity.^11–14^

The precise mechanism regulating HO-1 induction by heme or tissue erythrocytes had not been fully elucidated.^1^ This is surprising, given the intensive investigation of HO-1 and the physiological importance of its regulation by heme.^15^ We have characterized Mhem in human macrophages in vitro and human atherosclerotic intraplaque hemorrhage tissues.^16^ Mhem denotes a homeostatic macrophage phenotype distinct to M2, dependent on AMPK (AMP-activated protein kinase) and transcription factors ATF1 (activating transcription factor-1) and NFE2L2 (nuclear factor of erythroid cell 2-like 2).^16–18^ These mediate heme-stimulated induction of *HMOX1* via a cAMP response element–like/antioxidant response element–like (CRE [cAMP response element] like/ARE [antioxidant response element] like) cosite at −4200 bp.^16–20^ We define here the causal role of AMPK and ATF1 in the regulation of the normal process of hematoma resolution in vivo.

In a parabiosis model, ischemia-reperfusion injury modulates intracerebral hemorrhage resolution via AMPK, which was thought most likely to be mediated by transferred leukocytes.^21^ Here, we here define a direct and local intrinsic hemorrhage-resolution mechanism, driven via an AMPK-ATF1 pathway by the hematoma itself.

We show here that AMPK and ATF1 are required for normal hematoma resolution in vivo. Loss of either AMPK or ATF1 prolonged hematoma resolution, resulting in increased inflammation, protracted iron deposition, and increased oxidative tissue injury. ATF1 and AMPK mediated the dynamic regulation of gene expression by heme, including genes that coordinate erythrocyte disposal and tissue repair genes. Thus, ATF1 is a novel and important gene for macrophage-mediated tissue homeostasis.

## Methods

This article adheres to Transparency and Openness Promotion (TOP) guidelines. The data that support the findings of this study are available from the corresponding author upon reasonable request, and the transcriptomic data will be publicly available once formatted and accepted at the Gene Expression Omnibus (GEO).

More detailed Methods are available in the Data Supplement.

### Cell Culture

Bone marrow macrophages were cultured by a minor modification of previous methods. The mouse was sacrificed by schedule 1 method, and long bones cleaned sufficiently and flushed with ice-cold PBS as promptly as possible. The bone marrow is then cultured in 10% FCS Iscove Modified Dulbecco Medium supplemented with 10% L929-conditioned medium. At 6 days culture, the bone marrow macrophages were scraped and transferred to tissue culture plates optimized for experiments, typically 24-well plates.

### Reverse Transcription-Quantitative Polymerase Chain Reaction

RNA was purified by a proprietary kit (Qiagen). Cell culture supernatant was decanted and stored at −80°C. Then 200 μL guanidinium-based buffer (RNA lysis treatment) was added, the lysates stored at −80°C until purification (up to several weeks). Then the Qiagen mini column system was used for purification according to manufacturer’s instructions.

### Microarray and Bioinformatics

This was by minor modification of previous methods, detailed in the Data Supplement.^16^ RNA was quality controlled, labeled, and measured using the Agilent 4×44K mouse system, with a commercial service, Oxford Genome Technology (OGT). The data were analyzed with GeneSpring, with significance decided at a false discovery rate of 0.05 and Storey bootstrapping adjustment for multiple simultaneous comparisons. Gene Ontology was examined within GeneSpring and exported with the gene lists. Gene lists were analyzed outwith GeneSpring using standard packages, including Venn diagrams, GeneSet Enrichment Analysis. Gene names of characterized human and mouse genes are identical except for case. For human/mouse comparison, the cases were matched using Notepad++ and then submitted to Venn. Transcription factor–binding analysis was primarily using X2K (Ma’ayan Laboratory), which used ChIP (chromatin immunoprecipitation)-validated data for matching. Transcription factor motif searches with other methods broadly corroborated (named PASTAA [Predicting Associated Transcription Factors From Annotated Affinities], OPOSSUM, LASAGNA [Length-Aware Site Alignment Guided by Nucleotide Association], MEME [Multiple Em for Motif Elicitation], DREME [Discriminative Regular Expression Motif Elicitation], and TRANSFAC [Transcription Factor Database]). There was no time course on which to use time series–related methods of network inference, in contrast to our previous publications.^16^ Search Tool for the Retrieval of Interacting Genes/Proteins (STRING) (https://www.expasy.org/, Swiss Institute for Bioinformatics), which incorporates largely protein-protein binding but also other interactions, was used to construct networks, and the most highly connected nodes were identified.

### Femoral Hematoma

**Figure 1.**
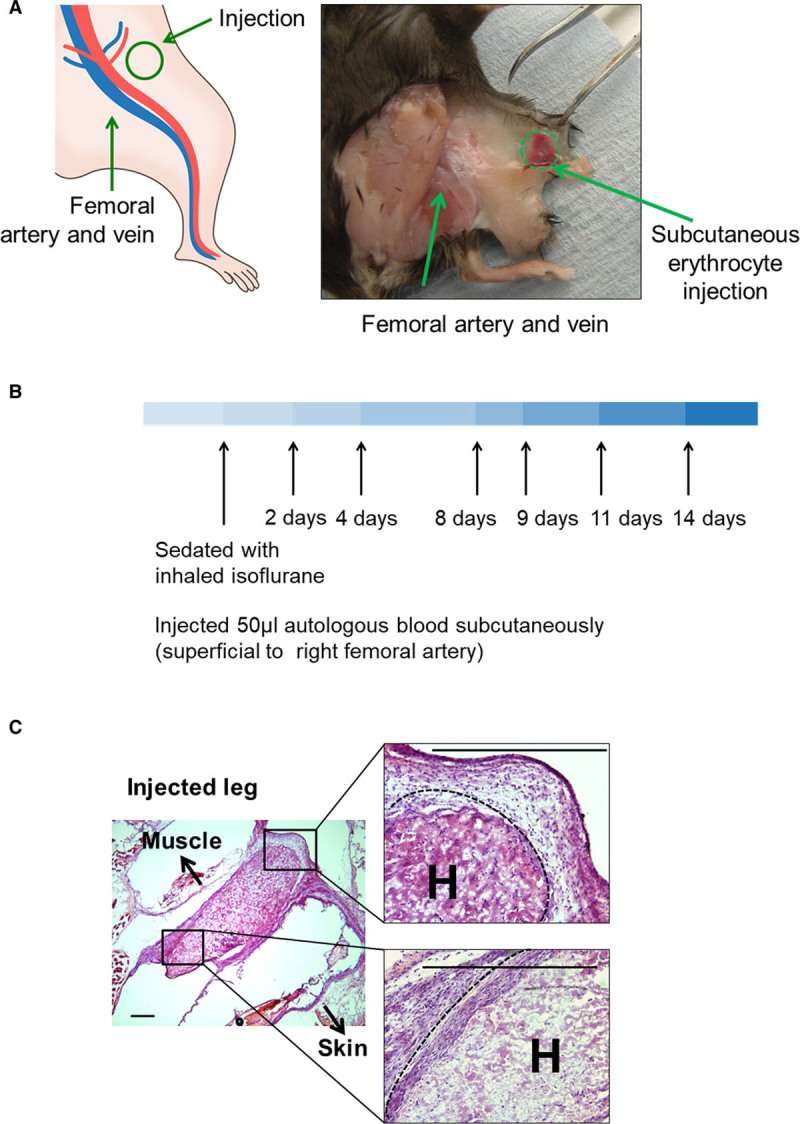
**Hematoma model.** **A**, Diagram (**left**) and photograph of a representative dissection (**right**) of the site of subcutaneous injection of littermate autologous blood (50 μL) into perifemoral adipose tissue. **B** shows the time course of the model, and **C** shows histological micrographs taken immediately after injection, showing topography. Arrows point in the direction of skin and muscle. A dashed line marks the edge of the hematoma. Scale bars=100 μm. H indicates injected hematoma.

Knockout and littermate control mice^22,23^ were lightly anesthetized with isoflurane to maintain humaneness and precision. Then autologous erythrocytes as 50 μL anticoagulated blood were injected into the femoral canal region (Figure 1). A preliminary time course indicated that there were residual hematomas at day 8, but these had cleared at day 9. Subsequent experiments focused on events at days 8 and 9.

### Microscopy and Confocal Microscopy and Image Analysis

Cryosectioning was at 5 μm using a Bright cryostat and specimens frozen in optimal cutting technology on cooled isopentane. Fluorescence-labeled sections were mounted in glycerol/PBS or in commercial antifade mountant and imaged within a few days on a Zeiss LSM750 (Imperial College Facility for Imaging by Light Microscopy). Standard UV/blue, 488, 560, and 750 channels were used, with additional differential interference contrast on the 488 channel. This instrument was also used for the fluorescence emission scans. Paraffin sectioning was at 5 μm, and histochemistry and immunohistochemistry is described in the Data Supplement.

### Optical Quantification

A Tecan Spectrafluor 96-well reader was used to acquire UV-visible absorption spectra and for the multiwavelength fluorescence excitation/emission combinations.

### Statistics

More detailed statistical methods are given in the Data Supplement. Data were analyzed with SigmaStat, GraphPad Prism, and SPSS. Data were tested for normality by Shapiro-Wilk where n ≥5. Where appropriate, data are given as mean±SE. Where appropriate, Student *t* test, Mann-Whitney *U*test, repeated measures ANOVA, or Kolmogorov-Smirnov were used where specified. Only in-test corrections were made. Where *P* values are not given, the testing was not performed.

## Results

**Figure 2.**
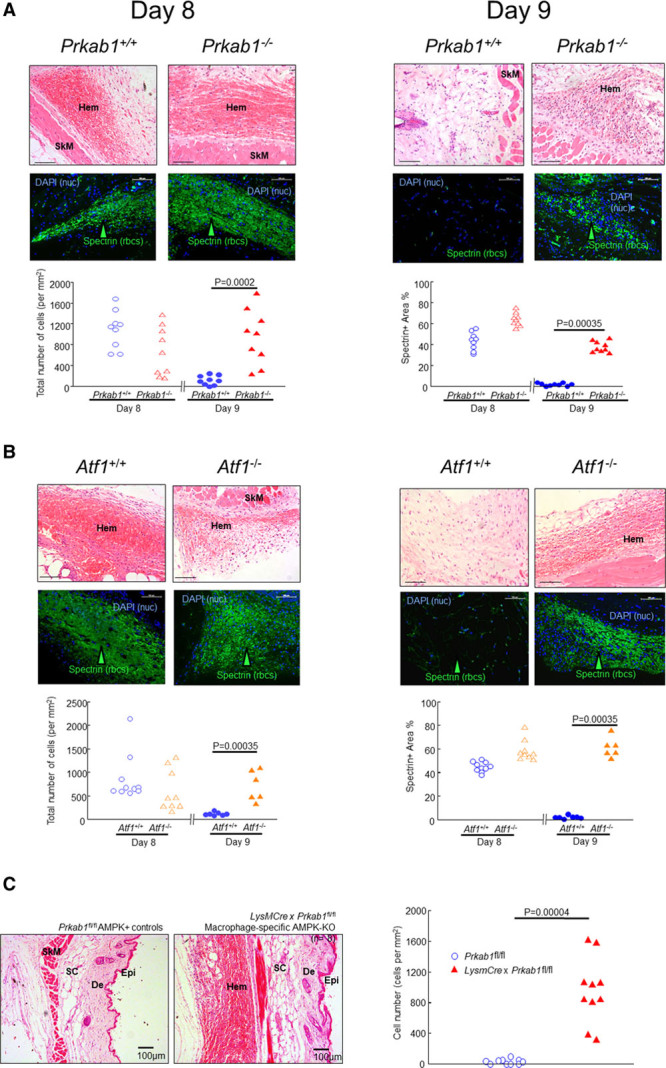
**Loss of AMPK (AMP-activated protein kinase) or ATF1 (activating transcription factor-1) delays hematoma (Hem) clearance**. The photomicrographs are representative of images of Hems obtained at day 8 or day 9 from mice with genetic deficiency of (**A**) AMPK (*Prkab1*^−/−^; n=9) or (**B**) ATF1 (*Atf1*^−/−^; n=6) or their littermate WT (wild type) controls (*Prkab1*^+/+^, *Atf1*^+/+^; n=9 and 9, respectively). The **top** rows show Hematoxylin and eosin (H&E) staining (scale bars=100 μm) and **bottom** rows show immunostaining with anti-spectrin antibodies (ie anti-erythrocyte) labeled with Alexa-488 (green) as indicated, arrowheads. Nuclei are stained blue with DAPI (4′,6-diamidino-2-phenylindole) to give total number of cells as indicated. Scale bars 100=μm. H&E (cell number) and immunostaining data are shown in the left- and right-hand graphs, respectively. The data points represent individual mice. Exact *P* values are as indicated for the indicated comparisons selected a priori (Mann-Whitney *U*test). Lineage specificity of the deletion is shown in **C**. The *Prkab1*-KOMP-Ko-first-conditional-possible allele was first crossed with *Frt*-mice to remove the STOP (STOP codon), yielding *Prkab1*−^fl/fl^. These were then crossed again to remove the *Frt*. The progeny with the crossed with *LysMCre* and the F1 double-het progeny intercrossed, yielding *LysMCre*×*Prkab1−*^*fl/fl*^ double homozygotes. These were then selected and bred as homozygotes. These Mac-AMPK-KO mice were then studied in the same Hem model as before. In the model, mice had Hems as expected at the earlier time point d8 (not shown, n=4). **Left**, Floxed controls without the Cre driver did not have Hems at the later time point (d9, n=9), indicating that these all cleared Hems as quickly as the other WT (wild type) controls. In contrast, mice lacking myeloid AMPK (*LysMCre*×*Prkab1*^*fl/fl*^) consistently had Hems still present at day 9 (10/10 mice, n=10; **right**). This indicates that the lack of Hem clearance in this model is specific to loss of AMPK in the macrophage lineage. Scale bars=100 µm. Graph, genotypes as indicated, each point represents 1 mouse. Exact *P*, Mann-Whitney *U test*. De indicates dermis; Epi, epidermis; KO, knockout; KOMP, Knockout Mouse Project; nuc, nuclei; rbcs, red blood cells; SC, subcutis; and SkM, skeletal muscle.

A subcutaneous hematoma model was developed as an experimentally reproducible and practical simulation of clinical hematoma (Figure 1A). Littermate autologous blood (50 μL) was injected into perifemoral adipose tissue (Figure 1A). Then, mice were sacrificed at set intervals (Figure 1B). The injected region was excised postmortem and examined by Hematoxylin and eosin (H&E) or immunocytochemistry (Figure 1C). Hematomas in genetic control mice consistently resolved between days 8 and 9 (*Prkab1*^+/+^ or *Atf1*^+/+^ littermate controls in Figure 2A and 2B). In contrast, mice deficient in AMPK (*Prkab1*^−/−^; Figure 2A) or ATF1 (*Atf1*^−/−^; Figure 2B) showed delayed hematoma resolution, as evidenced by the continued presence of blood at day 9. Specifically, residual erythrocytes were identified in both *Prkab1*^−/−^ and *Atf1*^−/−^ mice following staining with H&E or anti-spectrin antibodies (Figure 2A and 2B). Next, hematoma resolution in *LysMCre*×*Prkab1*^fl/fl^ mice was examined. This strain has AMPK selectively deleted from their macrophages and exhibited the same effect. That is, they retained hematomas at day 9, whereas *Prkab1*^fl/fl^ controls did not (Figure 2C). This indicated that it was macrophage AMPK that was required. To test further that the AMPK-ATF1 pathway was located in myeloid cells, a bone marrow transplant experiment was performed. In this, *Atf1*^+/+^ mice were reconstituted with marrow from *Atf1*^−/−^ or *Atf1*^+/+^ mice using standard bone marrow transplant procedures. *Atf1*^+/+^→*Atf1*^+/+^ cleared hematomas at day 9, and *Atf1*^−/−^→*Atf1*^+/+^ had residual hematomas at day 9 (Figure I in the Data Supplement). This indicated that the deficiency in clearing hematomas was transferred with *Atf1* deficiency in the hematopoietic lineage.

A number of validation assessments were also made. It was necessary to reversibly anticoagulate the blood to reproducibly transfer it to the recipients. This was done with a limiting concentration of citrate, which allows recalcification and resumption of coagulation on injection to tissue. The effect of this was determined by comparing hematomas with untreated and anticoagulated blood. Anticoagulation caused a slowing of the resolution (Figure IIB in the Data Supplement). A fuller time course was carried with the anticoagulated protocol, as it was more reproducible. This showed that hematomas appeared to be consistently maintained up day 8 but were lost between days 8 and 9 (Figure IIB in the Data Supplement). Measurement of the serum inflammatory marker SAA (serum amyloid A) indicated that the hematomas did not alter systemic inflammation (Figure IIC in the Data Supplement). Extending the experimental time to 21 days showed that in Atf1^−/−^ mice, hematoma clearance was simply delayed and the tissue returned to normal at day 21 (Figure IID in the Data Supplement).

No statistically significant difference was observed between hematocrits in *Atf1*^−/−^ and *Atf1*^+/+^ littermates (Figure IIIA in the Data Supplement). Spleen weights and spleen macrophage HO-1 expression were assessed in *Atf1*^−/−^ and *Atf1*^+/+^ littermate controls. Spleens of *Atf1*^−/−^ mice had a slightly lower mass than *Atf1*^+/+^ littermate controls and *Atf1*^−/−^ mice (Figure IIIB in the Data Supplement). *Atf1*^−/−^ mice and *Atf1*^+/+^ littermate controls had an equivalent CD68 (cluster of differentiation-68)+HO-1+ macrophage population in the splenic red pulp (Figure IIIC and IIID in the Data Supplement). *Atf1*^−/−^ mice and *Atf1*^+/+^ littermate controls had indistinguishable patterns and levels of splenic red pulp iron, which was located in splenic red pulp macrophages (RPMs; Figure IIIE and IIIF in the Data Supplement).

**Figure 3.**
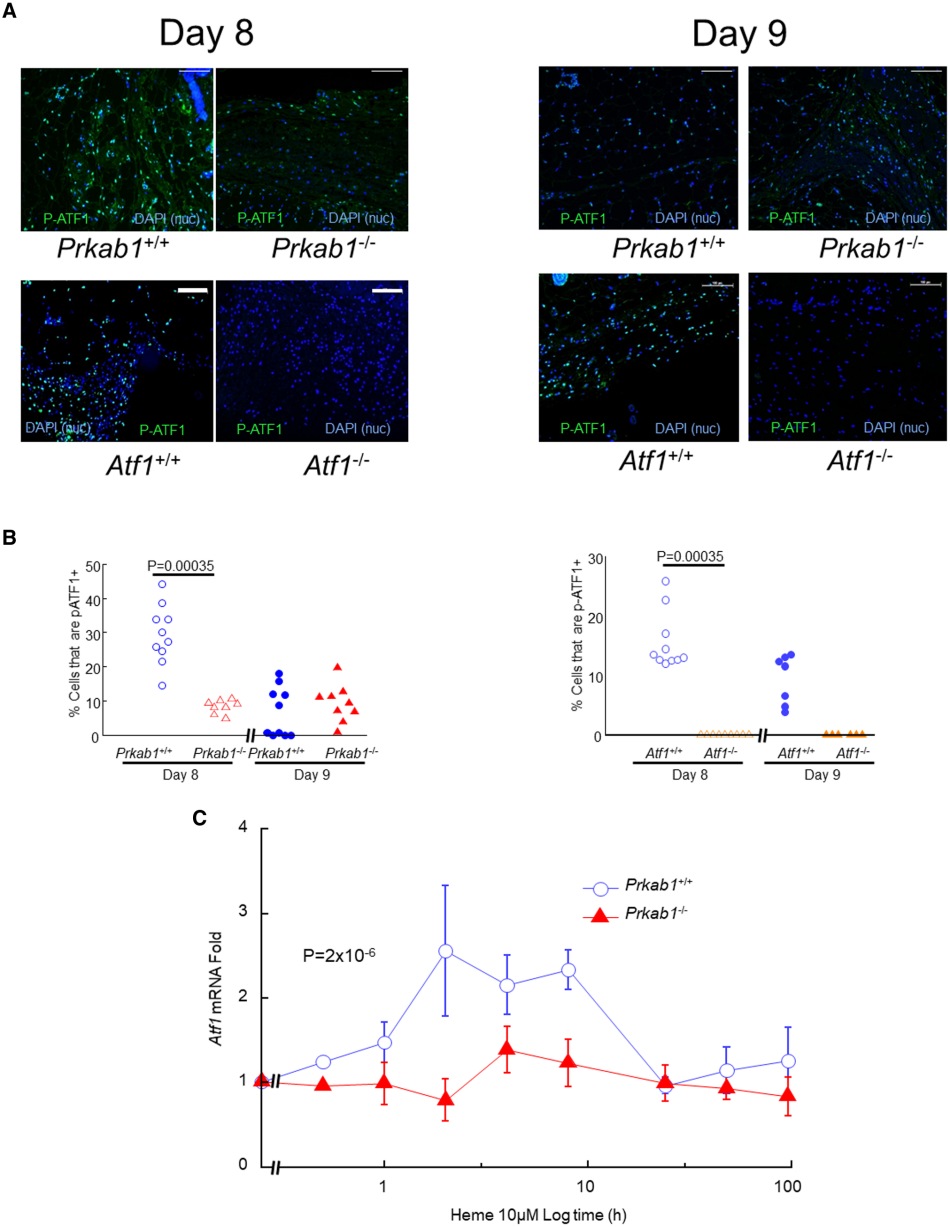
**AMPK (AMP-activated protein kinase) deficiency prevents ATF1 (activating transcription factor-1) induction.** **A**, Representative images of hematomas obtained at day 8 or day 9 after injection, after immunostaining green for phosphorylated ATF1 (pATF1) as indicated. The **top** row shows *Prkab1*^+/+^ (n=9) vs *Prkab1*^−/−^ (n=9) mice, and the **bottom** row shows *Atf1*^+/+^ (n=9) vs *Atf1*^−/−^ (n=6). Scale bars=100 μm. Nuclei are stained blue with DAPI (4′,6-diamidino-2-phenylindole) as indicated. **B**, Image quantification of the effects of AMPK and ATF1 deficiency are shown in the left- and right-hand graphs, respectively. Data points are individual mice. Exact *P* values are indicated for indicated comparisons selected a priori (Mann-Whitney *U*test). **C** shows the failure of *Atf1* mRNA induction in *Prkab1*^−/−^ macrophages in vitro. Bone marrow–derived macrophages (mBMMs) from *Prkab1*^−/−^ or WT (wild type) littermate controls were stimulated by 10 μM heme and cells harvested after varying durations for assay of mRNA transcripts by reverse transcription-quantitative polymerase chain reaction. The *y* axis is fold induction relative to vehicle, calculated using the −ΔΔC_t_ method. The *x* axis is time using a log scale to facilitate plotting of both rapidly changing and slowly changing expression. Exact *P* given for repeated measures 1-way ANOVA. Data points are mean±SE, n=6, values in Supplemental. Nuc indicates nuclei.

Immunolabeling of day 8 hematomas from AMPK-deficient mice (*Prkab1*^−/−^) for phosphorylated ATF1 (pATF1) showed significantly less pATF1 expression than hematomas from genetic control mice (Figure 3A). This indicated pATF1 was dependent on AMPK. The null labeling in *Atf1*^−/−^ mice confirmed the specificity of the antibody (Figure 3A). Image quantification showed that lesional pATF1 was consistently profoundly suppressed in *Prkab1*^−/−^ mice and absent in *Atf1*^−/−^ mice (Figure 3B). As shown in Figure 3C, induction of the *ATF1* gene by heme in cultured mouse BM-derived macrophages was profoundly reduced by AMPK deficiency. This indicated that ATF1 transcriptional activation is AMPK dependent. These observations, combined with our previous data on human macrophages in vitro,^17^ indicate that ATF1 expression and phosphorylation due to heme are dependent upon AMPK.

**Figure 4.**
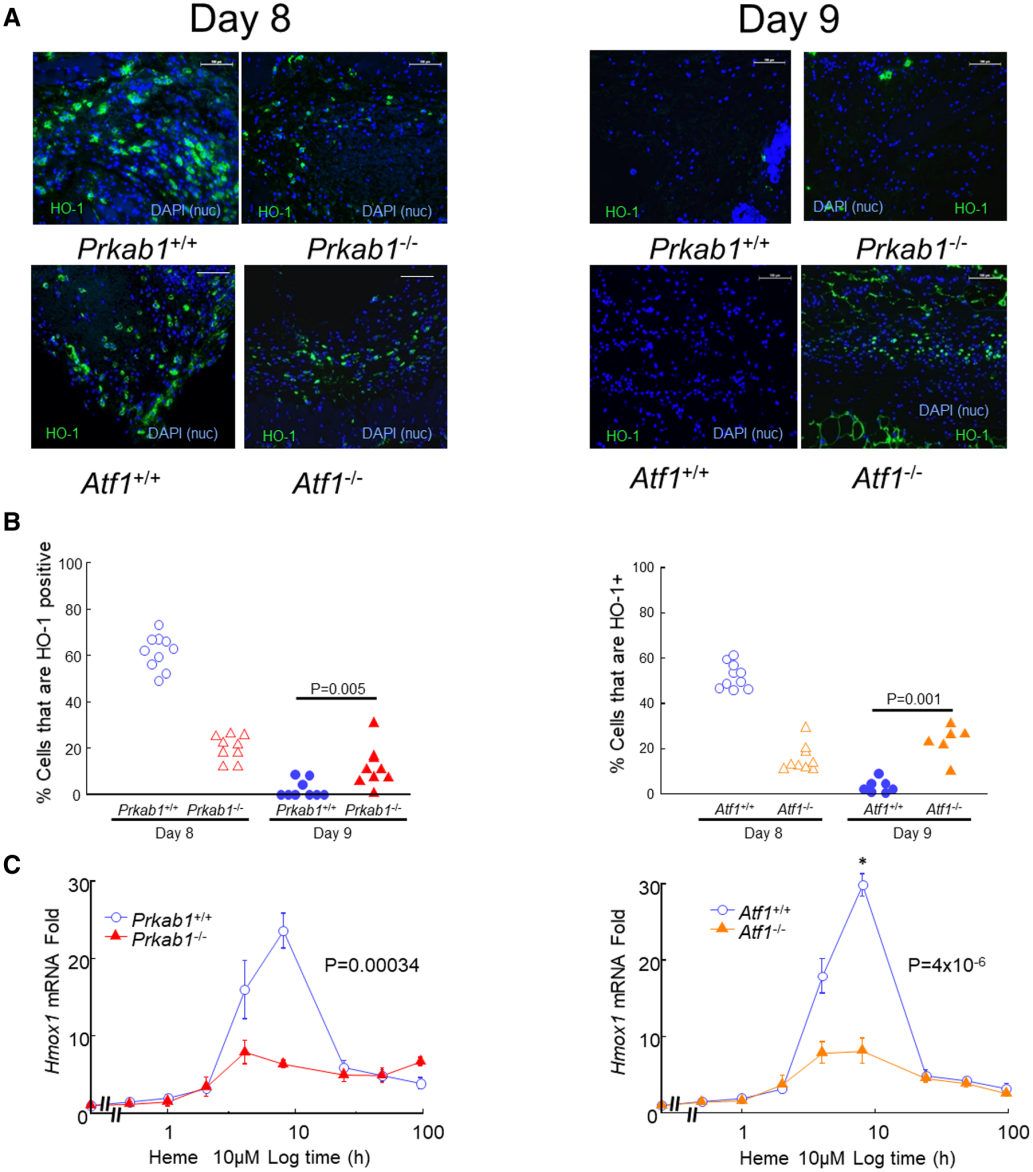
**AMPK (AMP-activated protein kinase) or ATF1 (activating transcription factor-1) deficiency prevents HO-1 (heme oxygenase-1) expression in hematomas**. Hematomas obtained at day 8 or day 9 after injection were immunostained green for HO-1 as indicated. **A**, Representative images. The **top** row shows *Prkab1*^+/+^ (n=9) vs *Prkab1*^−/−^ (n=9) mice, and the **bottom** row shows *Atf1*^+/+^ (n=9) vs *Atf1*^−/−^ (n=6). Scale bars=100 μm. Nuclei are stained blue with DAPI (4′,6-diamidino-2-phenylindole) as indicated. **B**, Image quantification of the effects of AMPK and ATF1 deficiency are shown in the left- and right-hand graphs, respectively, based on images in **A**. Data points are individual mice. Exact *P* values are shown for the indicated comparisons selected a priori (Mann-Whitney *U*test). **C** shows the impact of AMPK or ATF1 deficiency on transcriptional activation of *Hmox1* in mBMM stimulated with heme (10 μM). Exact *P* given for repeated measures 1-way ANOVA. See Figure 3 legend for more methodological detail. Data points are mean±SE, n=6, values in Supplemental. Nuc indicates nuclei.

**Figure 5.**
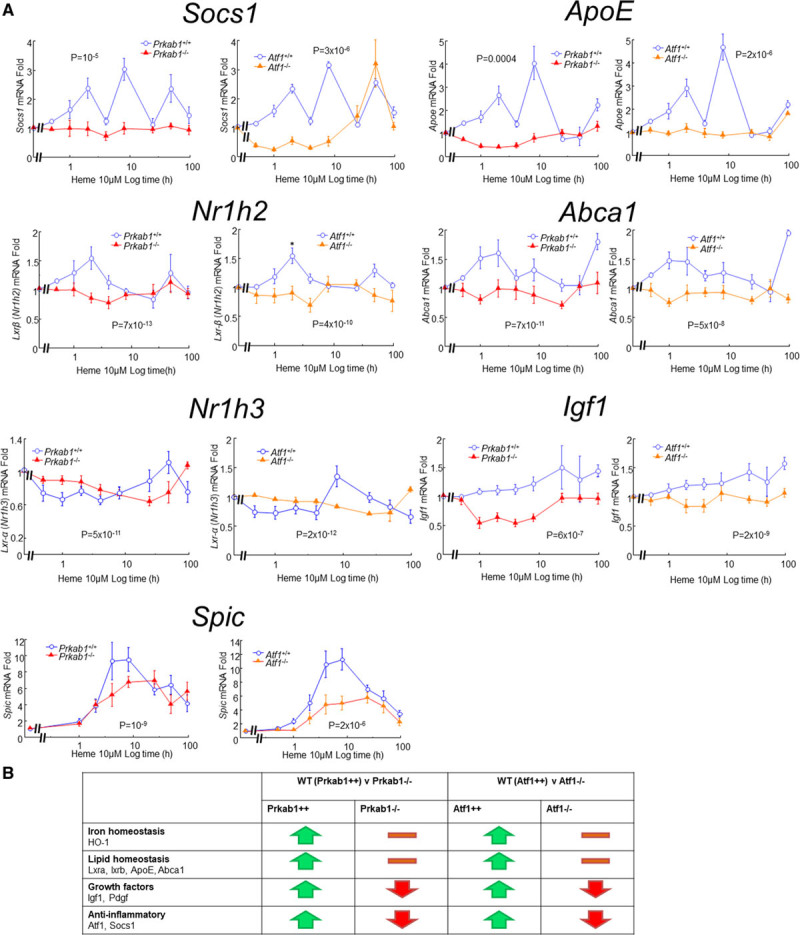
**Heme-regulated gene expression in mBMM and effects of AMPK (AMP-activated protein kinase) or ATF1 (activating transcription factor-1) deficiency.** **A**, mBMMs from *Prkab1*^−/−^, *Atf1*^−/−^, or WT (wild type) littermate controls were stimulated in vitro by 10 μM heme and cells harvested after varying durations for assay of mRNA transcripts by reverse transcription-quantitative polymerase chain reaction, as in Figure 3B. Data points are mean±SE, n=6; Exact *P* given for repeated measures ANOVA. **B**, Summary of the impact of AMPK and ATF1 deficiency, which had very similar effects. Abca1 indicates ATP-binding cassette family subfamily a member 1; ApoE, apolipoprotein E; HO-1, heme oxygenase-1; Igf1, insulin-like growth factor-1; Lxr, lipid X receptor; Pdgf, platelet derived growth factor; Prkab1, protein kinase AMP-activated beta subunit 1; and Socs1, silencer of cytokine signaling 1.

We have previously reported that *HMOX1* is an ATF1 target gene in human macrophages exposed to heme.^16^ This was also true of mice. We found impaired expression of HO-1 in day 8 hematomas (HO-1) from *Atf1*^−/−^ mice (*P*=0.001; Figure 4A and 4B) and significantly reduced *Hmox1* gene expression in heme-stimulated *Atf1*^−/−^ (*P*=4×10^−6^; Figure 4C). Consistent with the dependency of ATF1 on AMPK, similar data were obtained with hematomas from *Prkab1*^−/−^ mice in vivo and with *Prkab1*^−/−^ bone marrow–derived macrophages in vitro (Figure 4A through 4C). The time course of gene expression in response to 10 μM heme was also assessed for a number of other genes potentially affected by AMPK or ATF1 deficiency (Figure 5). We found that AMPK and ATF1 deficiency had similar effects on expression of heme-responsive genes, including *Hmox1*, *Socs1*, *Nr1h2* (LXR [lipid X receptor]-β), *Nr1h3* (LXRα), *Abca1*, *Apoe*, and *Igf1* (Figure 5A). The gene *Spic* was also *Atf1* dependent (Figure 5A). Heme-dependent commitment to splenic RPMs is mediated by *Spic*,^24^ indicating that *Atf1* may also be above *Spic* in the hierarchy of heme-dependent regulation more generally. Taken together with the in vivo data presented above, these results support an AMPK/ATF1 pathway regulating heme-induced homeostatic genes involved in iron metabolism, lipid metabolism, and inflammation (Figure 5B).

**Figure 6.**
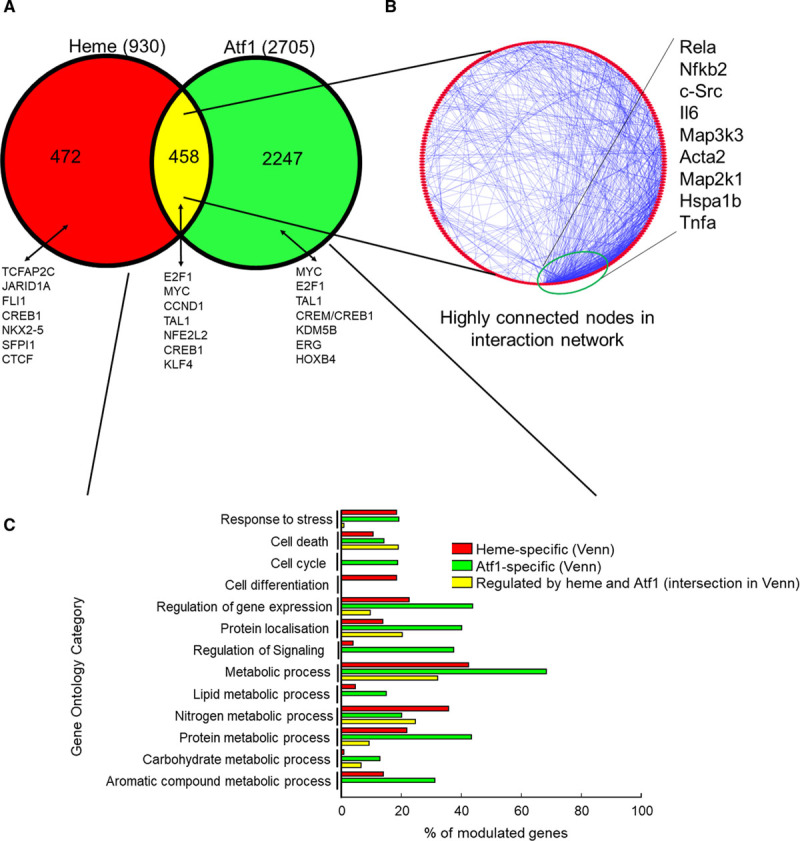
**Genomic responses to heme in ATF1 (activating transcription factor-1)-deficient compared with WT (wild type) mice.** Microarrays were used to interrogate gene expression in mBMM stimulated by heme for 8 h, comparing cells from *Atf1*^−/−^ and littermate *Atf*^+/+^ mice. **A**, Venn diagram showing numbers of genes regulated by heme or ATF1. Overrepresented transcription factor–binding sites (TFBSs) are shown for each section. **B**, Network analysis of mouse genes regulated by both heme and *Atf1*. The most connected nodes are shown and point to immune and stress response drivers as key hubs for the Atf1-dependent response to heme. **C**, Gene ontology analysis of genes from **A**, colored by whether they reflect gene sets in the heme-specific (red), *Atf1*-specific (green), or heme- and *Atf1*-coregulated (yellow) sections of the Venn diagram in **A**.

We extended the assessment of gene regulation by using microarrays in an unbiased manner across the entire transcriptome. This allowed us to compare human and mouse heme-induced responses and to evaluate the role of ATF1 and other transcription factors. Based on the quantitative polymerase chain reaction data above, we chose the 8-hour time point to compare responses to heme versus vehicle in *Atf1*^−/−^ and *Atf1*^+/+^ WT (wild type) macrophages (Online Video I [Omics Dataset]). Approximately one-half of the heme-regulated genes (458 of 930) were regulated by heme in an *Atf1*-dependent manner (Figure 6A). Identification of overrepresented transcription factor–binding site in gene sets in each category showed increased sites for KLF4 (Krüppel-like factor 4), ATF1/CREB1 (CRE-binding protein isoform)-1, and NFE2L2 (NRF2) in the heme- and *Atf1*-regulated genes (Figure 6A). The enrichment in transcription factor–binding site for bZIP (basic Zipper) members—specifically CREB/ATF1, AP-1 (activator protein 1), and NFE2L2/Bach/MafG (musculoaponeutotic fibrosarcoma oncogene homolog G)—was similar to humans.^16^ The enrichment for KLF4 sites was intriguing since KLF4 has been linked to other proresolution prorepair homeostatic differentiation patterns in macrophages.^25,26^ Next, taking the heme- and *Atf1*-regulated genes and examining their interactions in the public protein-protein binding database STRING revealed that highly connected regulators were known key inflammatory genes including NFκB (nuclear factor-kappa B; *Rela*), IL6 (interleukin-6), TNF-α (tumor necrosis factor-alpha), and members of the MAPK (mitogen-activated protein kinase) family (Figure 6B). This was consistent with heme modulating inflammation via *Atf1*.

Next, the genes in the 3 areas of the Venn diagram (Heme-only, *Atf1*-only, and heme-*Atf1* coregulated intersection) were compared by Gene Ontology classification (http://www.pantherdb.org/). This revealed similarities and also substantial differences between the three sets of genes (Figure 6C). Broadly, they modulated cell stress, cell death, division, and differentiation. Many of the genes were involved in metabolic processes (lipid, nitrogen, protein, carbohydrate, aromatic compound). At a more detailed level, there were substantial differences between the three pathways. Thus, the heme-*Atf1* coregulated intersection corresponded to fewer stress genes, the *Atf1*-specific section to more cell-cycle genes and to regulation of signaling, and heme-specific genes more to cell differentiation (Figure 6C).

Of note, the next most frequent group of Gene Ontology terms was the relatively small number corresponding to immune regulation (not shown). While all 3 pathways modulated immune regulatory genes, they modulated distinct categories. Thus, genes modulated by heme and *Atf1* had 13% of genes corresponding to Gene Ontology term lymphocyte activation; 2.3% of heme-specific genes to regulation of lymphocyte-mediated immunity and 0.61% of genes modulated by Atf1 only corresponded to immunoglobulin-mediated response, with zero value for the remainder (not shown). This indicates that although the three pathways modulated inflammation/immunity, they did so in patterns with alternative biological fine-tuning.

**Figure 7.**
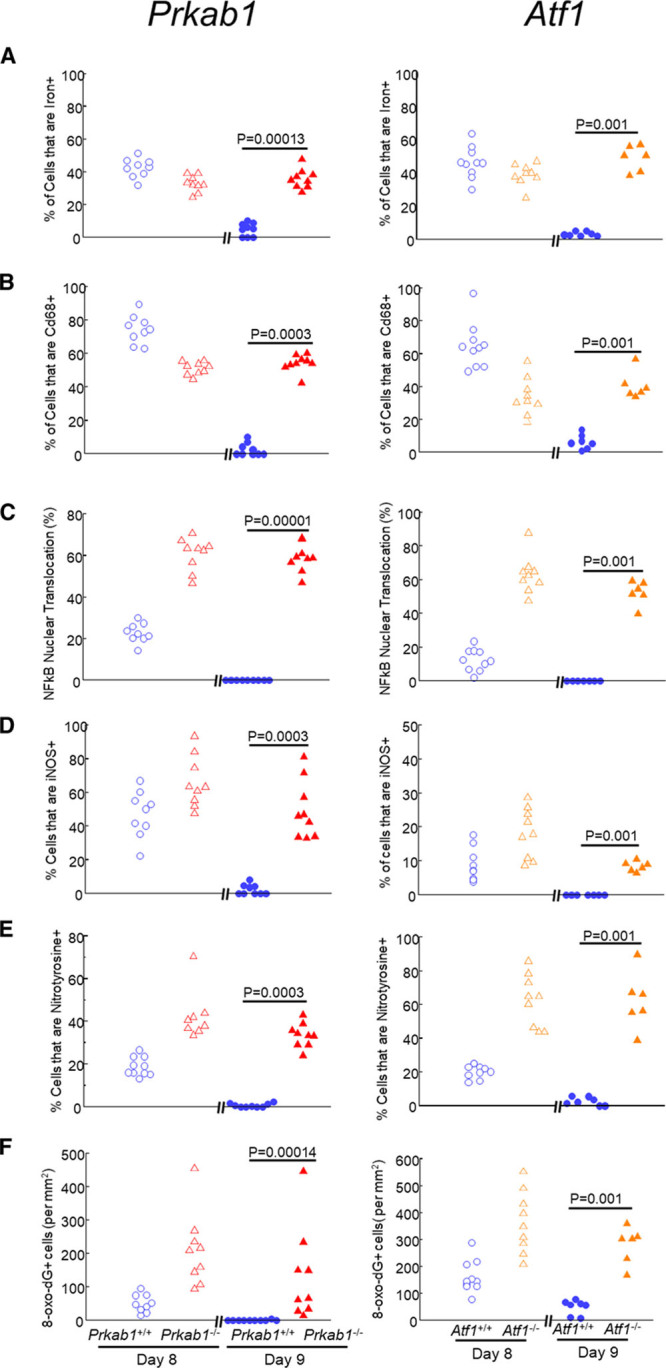
**Prolonged inflammatory activation and tissue injury in the absence of AMPK (AMP-activated protein kinase) or ATF1 (activating transcription factor-1)**. Hematomas were obtained at day 8 or day 9 after injection from *Prkab1*^−/−^ (left-hand graphs, n=9) or *Atf1*^−/−^ (right-hand graphs, n=6), together with *Prkab*^+/+^ (n=9) or *Atf1*^+/+^ (n=9) littermate WT (wild type) controls. Sections were stained (**A**) with Perl iron stain (Prussian blue), with nuclear counterstaining by nuclear fast red or immunostained (green) for (**B**) macrophages (CD68 [cluster of differentiation-68]); (**C**) *p65 Rela* (NFkB), with quantification of percentage nuclear translocation; (**D**) iNOS (inducible NO synthase; *Nos2*); (**E**) nitrotyrosine; and (**F**) 8-oxo-deoxyguanosine. Data points are individual mice. Exact *P* are as indicated for the indicated comparisons (Mann-Whitney *U* test).

**Figure 8.**
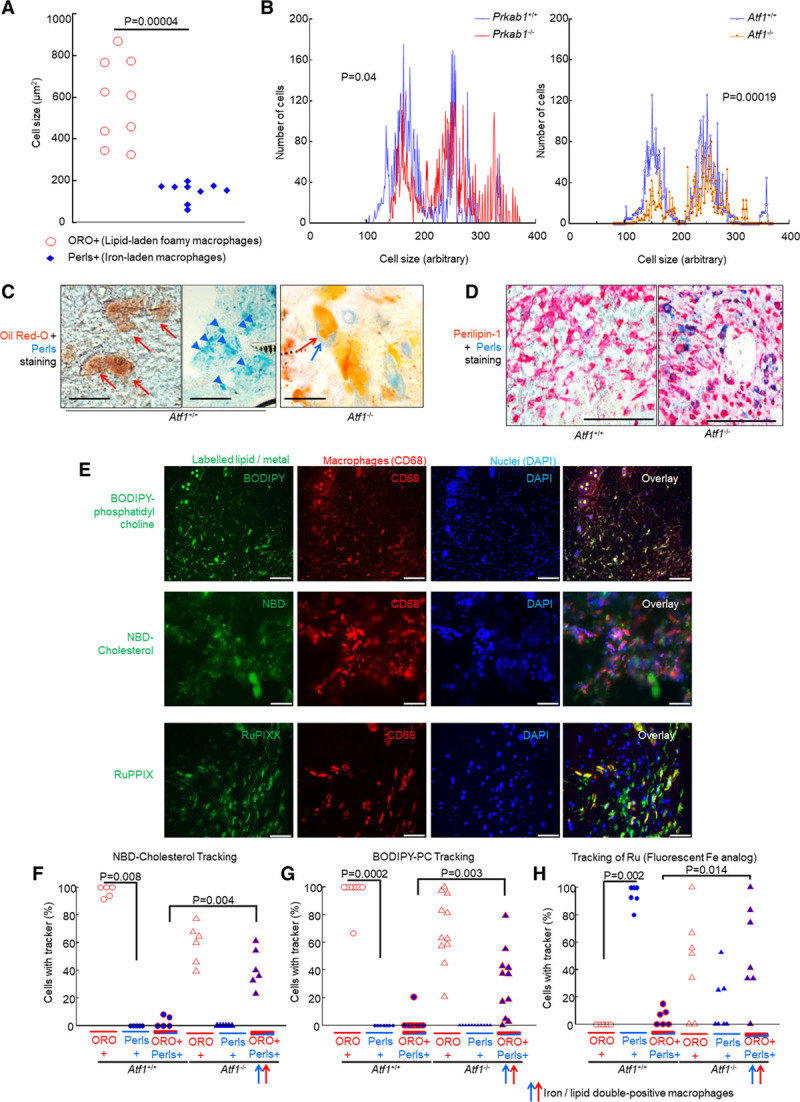
**AMPK (AMP-activated protein kinase) and ATF1 (activating transcription factor-1) are required for divergent differentiation into iron-laden macrophages and lipid-laden macrophages, reflecting trafficking of iron and lipid from erythrocytes, respectively, into iron-laden macrophages or lipid-laden macrophages.** **A**, Relative sizes of foamy macrophages (lipid-laden, Oil Red O [ORO] stain) and hemosiderin-laden macrophages (iron-laden, Perl stain) in resolving mouse hematoma, from a normal mouse at day 8. The absolute cell areas are shown on the *y* axis. Each point represents 1 mouse (n=9). *Exact *P*, Mann-Whitney *U*test. **B**, Size distribution of macrophages (*Cd68+*) and effect of knockout status, showing a bimodal distribution, indicating that there are 2 populations, that is, small and large macrophages. *Kolmogorov-Smirnov test, small macrophages are fewer in either *Atf1*^−/−^ (*P*=0.00019) or *Prkab1*^−/−^ (*P*=0.04). Data obtained by histological image cytometry. **C**, Representative micrographs, effect of loss of *Atf1* on separation of lipid and iron. Dual stained with Berlin Blue and Oil Red O (red, neutral lipid; blue, iron). **Left**, Iron and fat are found in separate cells in *Atf1*^+/+^. **Right**, Iron and fat are found in the same cell in *Atf1*^−/−^. Scale bars=50 μm. **D**, Paraffin sections of hematomas at day 8, stained with Berlin Blue histochemistry combined with immunohistochemistry for perilipin-1, an antigen induced in foam cells, stable in formalin-fixed paraffin-embedded tissues. Representative images, scale bars=100 μm. **Left**, Large perilipin-positive foam cells largely without iron, with small iron-positive cells in distinct zones (not shown). **Right**, Foam cells are far more strongly positive for iron in *Atf1*^−/−^ mice indicating increased colocalization of iron and lipid. **E**, Representative images of hematomas with metabolite tracking. Erythrocytes were injected containing the indicated label (boron dipyrromethene-phosphatidylcholine [BODIPY-PC], nitrobenzoxadiazole [NBD]-cholesterol, ruthenium [Ru]) and then imaged at 8 d, with immunostaining for Cd68 (cluster of differentiation-68) macrophage marker. **Right**, Overlay. Fluorescent markers are as indicated, colors approximate true color (green, ≈530–540 nm; red, ≈540–560 nm; blue, ≈460–480 nm). Right-hand column, overlay where yellow=green and red, indicating colocalization between respective metabolites and macrophages. Representative n=5 mice each. Scale bars=100 μm. **F–H**, Quantification of tracking of fluorescent lipid and metal to lipid-laden and iron-laden macrophage, in the presence and absence of *Atf1*. Exact *P* values are shown for the indicated comparisons (Mann-Whitney *U*test) selected a priori. The data passed Shapiro-Wilk, but in view of economical mouse numbers and skew on visual inspection, a nonparametric analysis was used. Only 2 analyses were critical to the hypothesis. **F**, Labeled cholesterol tracks into lipid-laden macrophages normally (*P=0.008, Mann-Whitney *U*test) but into an abnormal lipid/iron dual-positive macrophage population in *Atf1*^−/−^ (*P*=0.004, Mann-Whitney *U*test). **G**, Labeled phosphatidyl choline (PC; *P*=0.0002, Mann-Whitney *U*test), tracked into lipid-laden macrophages normally but into iron/lipid double-loaded macrophages in *Atf1*^−/−^. **H**, Ru, the fluorescent Fe tracker has the converse pattern, tracking into iron-laden macrophages normally (*P=0.002, Mann-Whitney *U*test) but into the abnormal lipid/iron dual-positive macrophages in *Atf1*^−/−^ (*P*=0.014, Mann-Whitney *U*test). DAPI indicates 4′,6-diamidino-2-phenylindole.

On the basis of these results, we next explored whether delayed blood clearance in *Prkab1*^−/−^ or *Atf1*^−/−^ mice was associated with disrupted homeostasis, oxidative/nitrosative stress, and tissue injury. As expected from the continued presence of erythrocytes, deficiency in either AMPK or ATF1 led to the persistence of Berlin Blue–stainable iron at day 9 (Figure 7A). We also found an increase in macrophage number, as evaluated by anti-CD68 immunolabeling (Figure 7B). This was associated with increased macrophage inflammatory activation, as reflected by increased NFκB *p65 Rela* cytoplasmic-to-nuclear translocation (Figure 7C), as well as increased expression of iNOS (inducible NO synthase; *Nos2*; Figure 7D). Protein and DNA modifications in the presence of iron and activated macrophages were evidenced by increased immunolabeling for nitrotyrosine (Figure 7E) and 8-oxo-deoxyguanosine (Figure 7F), respectively, indicating nitrosative and oxidative stress.

Macrophages engaged in hematoma clearance normally become hemosiderin-laden macrophages (siderophages) or lipid-laden macrophages (foamy macrophages, foam cells). Consistent with this, dual histochemistry with Oil Red O and Berlin Blue (Prussian blue, Perls stain—a well-characterized iron histochemistry reaction) demonstrated that iron was contained in small macrophages and lipid in large macrophages (Figure 8A). This size separation, measured as a bimodal size distribution, was disrupted in *Prkab1*^−/−^ and *Atf1*^−/−^ mice (Figure 8B). When this was addressed in more detail with Oil Red O–Berlin Blue dual histochemistry, there were separate iron-positive and lipid-positive macrophages in *Atf1*^+/+^ littermate controls, but iron and lipid were colocalized in the same macrophages in *Atf1*^−/−^ mice (Figure 8C). That is, loss of *Atf1* resulted in accumulation of lipid and iron in the same macrophages. The same pattern was seen in paraffin sections using the foam cell marker perilipin-1 and Berlin Blue (Figure 8D).

To probe causality, we next examined active tracking of metal and lipid metabolites from erythrocytes into the siderophages and lipid-laden macrophages, in the presence and absence of *Atf1*. The ATF1-mediated coregulation of lipid and iron homeostatic genes in response to heme provides a mechanism for separate differentiation into iron-laden macrophages and lipid-laden macrophages. Whether AMPK and ATF1 were required for separation into iron-laden macrophages and lipid-laden macrophages by directing active trafficking of metabolites was next tested using fluorescently labeled lipid and iron analogs. Boron dipyrromethene-phosphatidylcholine and nitrobenzoxadiazole-cholesterol served as tracking reagents, respectively, for phospholipid and cholesterol.^27^ In preliminary experiments, we validated ruthenium (Ru) as a fluorescent analog for iron tracking (Figures IV through LVI in the Data Supplement). Ru and Fe are in the same group in the periodic table, have approximately the same ionic radius, and Ru becomes fluorescent on complexing with the pyrrole nitrogen in porphyrins or histidine (in ferritin).^28^ Ru is not severely toxic.^29^ Erythrocytes containing Ru–protoporphyrin-IX or fluorescently labeled lipids were injected as above to create labeled hematomas. Fluorescently labeled lipids trafficked to large macrophages (Figure V in the Data Supplement). Fluorescence corresponding to Ru-ferritin was identified in small macrophages, indicating that the labeled erythrocyte-derived Ru–protoporphyrin-IX had been taken up, processed, and stored (Figure 8E). Loss of *Atf1* resulted in delayed clearance of Ru-associated fluorescence, with the appearance of Ru-associated fluorescence in cells that were large and lipid laden, corresponding to foam cells (Figure 8F through 8H).

We next asked whether the deficiency in *Atf1*^−/−^ for hematoma clearance was specific to erythrocytes or reflected a more generalized macrophage dysfunction. Clearance of apoptotic neutrophil leukocytes (efferocytosis) was, therefore, studied. Strain controls were sacrificed and bled. Neutrophils were purified from blood and then killed with UV irradiation (20 minutes), labeled for tracking, and then injected to the perifemoral area. After a time course, mice were sacrificed and the femoral area examined by classical histology, as for the hematomas. Deficiency in *Atf1* did not affect apoptotic leukocyte clearance (Figure VII in the Data Supplement). Conversely, apoptotic leukocyte clearance in this model was severely impaired by si-*Creb1* knockdown (not shown). *Creb1* canonically mediates transcriptional responses to cAMP—the second messenger used by PGE_2_ (prostaglandin E_2_) and specialized proresolving mediators that play an important role in inflammation.^30–32^

## Discussion

We show here that deletion of either ATF1 or AMPK impairs normal hematoma resolution in vivo, causing hematoma persistence with inflammation and oxidative stress. Heme modulated numerous genes via *Atf1*, including metabolic genes and genes for nitrogen-metabolism, cell death, protein localization, and gene expression. Fluorescent erythrocyte components tracked into macrophages, with metal tracking into siderophages and lipid into foamy macrophages. This separation was disrupted by *Atf1* gene deletion. Loss of *Atf1* induced inflammation, oxidative stress, and pathological colocalization of lipid and iron. *Atf1* is hierarchically upstream of *Spic*.

Heme modulated important clusters of genes. We found an *Atf1*-dependent and an AMPK-dependent induction of genes regulating growth, metabolism, and inflammation. This corresponded with the broader microarray data in which heme and *Atf1* modulated genes widely across metabolic regulation. We also established in vivo–in vitro and mouse-human correlations in which the previous in vitro data implicating AMPK in ATF1 activation were replicated in vivo. Hematoma pATF1 activation was prevented in AMPK knockouts, resulting in loss of *Hmox1* induction, with promotion of inflammation and oxidative stress.

We took care to establish that *Atf1* actively separated iron and lipid. We established that Ru would track Fe on a cell-by-cell basis—a necessary improvement on isotopic methods for lineage determination. We coupled this with cell size analysis, lipid tracing, and iron/lipid dual histochemistry to show that metal and lipid from erythrocytes are actively trafficked into different macrophages dependent on *Atf1*.

Much research emphasis in recent decades has been on resolution of inflammation, notably mechanisms of leukocyte clearance, more particularly disposal of apoptotic leukocytes (efferocytosis). In preceding decades, there had been interest in the resolution of hematomas and tissue hemorrhage. For example, a time course and eventual outcome of hematomas artificially injected in arterial walls was defined.^33^ Such studies allowed the definition of HO-1 as the enzyme activity that degrades heme and thereby mediates the classic color sequence of bruise resolution.^8,11,34,35^ The key inducible isoform of heme oxygenase was defined in the 1980s^36^ and sequenced in the 1990s.^37^ This article defines HO-1 regulation and coregulation in its original physiological context. Our future work will focus on understanding gene regulation during erythrocyte resolution relative to inflammation resolution, focusing on the specificity mechanisms between ATF1-related gene regulation and CREB1-related gene regulation.

The subcutaneous femoral hematomas resolved between days 8 and 9, which indicated a relatively abrupt clearance event. This was unexpected but facilitated robust model measurement and experimental data. This model, together with in vitro experiments, provided new insights into erythrocyte clearance. A more precise molecular understanding of the interregulation and specificity mechanisms of AMPK, CREB1, ATF1, and their target genes is a priority. A full follow-up of the dependency of *Spic* on *Atf1* was also outwith the scope of the present article. *Spic* mediates commitment to splenic RPM, apparently in response to heme.^24,38^ At face value, that would suggest that splenic RPM may fail to develop in the *Atf1*^−/−^. However, although the spleens in *Atf1*^−/−^ were of slightly lower weight, no statistically significant difference was observed in iron content or in histology or macrophage content of HO-1–positive macrophages. *Atf1* may, therefore, reflect a specifically dynamic system that adapts to transient high-level heme, while other signaling pathways to *Spic* mediate the constitutive differentiation of RPM.

Moreover, we restricted examination to one artificial model of hematoma. It may be that multiple diseases and disease models involving hemorrhage are modulated by the *Hmox1*-regulating function of *Atf1*. These may include advanced atherosclerotic plaques,^1^ intracranial hemorrhage,^39^ alveolar hemorrhage,^40^ ischemia-reperfusion injury,^41^ and neurodegeneration.^42,43^ Indeed, in humans, *ATF1* is a risk locus for sudden cardiac death in the context of coronary calcification.^44^ Unfortunately, there are no current good models for these, and we are in the process of developing these with collaborators. However, this model has clinical relevance given the complications of femoral hematoma post-angiography and primary coronary intervention. These include prolonged in-patient stay and indeed mortality, so are not minor problems.^45^ Predisposing factors include strength of anticoagulation, comorbidities, and age.^45^

This specificity was mirrored in vivo, with deficient erythrocyte clearance in *Atf1*^−/−^ mice (but not leukocyte clearance) while leukocyte clearance was deficient in si-*Creb1* knockdown. The specificity mechanism is unknown but potentially fascinating.

## Conclusions

AMPK and ATF1 are required for hematoma clearance in vivo. Their loss increases iron deposition, inflammation, and nitrosative and oxidative stress. ATF1 coregulates genes for hematoma clearance, anti-inflammatory genes, and *Spic*—the transcription factor that drives splenic RPM. Erythrocyte iron and lipid are systematically segregated into distinct macrophage populations dependent on *Atf1*. ATF1, therefore, plays a specific and important role in the normal resolution of tissue hemorrhage, which may be its principal function.

## Sources of Funding

This work was funded by a British Heart Foundation Senior Clinical Research Fellowship (FS/13/12/30037) to J.J. Boyle. Some of the imaging was performed with instruments in the Facility for Imaging by Light Microscopy (FILM), Imperial College London. FILM at the Imperial College London is, in part, supported by funding from the Wellcome Trust (grant 104931/Z/14/Z) and Botechnology and Biological Sciences Research Council (BBSRC) (grant BB/L015129/1). The work was also supported by the NIHR Imperial Biomedical Research Centre. The views expressed are those of the author(s) and not necessarily those of the National Institute for Health Research (NIHR) or the Department of Health and Social Care.

## Disclosures

None.

## Supplemental Materials

Expanded Materials & Methods

Online Figures I–VII

Online Video I (Omics Dataset)

References^16–19,22,23,46–59^

## Supplementary Material



## Novelty and Significance

### What Is Known?

- Hematomas occur during leakage of blood vessels and can contribute to cardiovascular morbidity or mortality in various settings such as stroke.
- Hematomas may also occur at sites of vascular access and be worsened in anticoagulated patients. Hematoma resolution occurs over time as the blood debris is removed.
- Heme is cleared via HO-1 (heme oxygenase-1).
