# Supplementary figures and images for: Hematoma Resolution In Vivo Is Directed by Activating Transcription Factor 1
Source: Circ Res. 2020 Jul 2;127(7):928–44. doi: 10.1161/CIRCRESAHA.119.315528 (PMC7478221; doi:10.1161/CIRCRESAHA.119.315528)

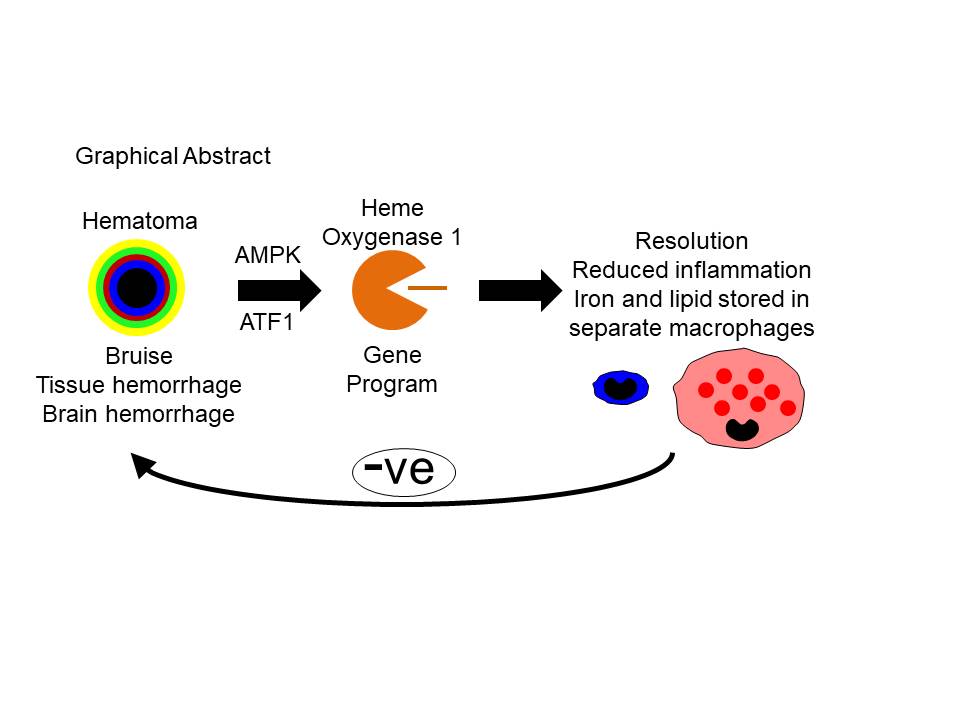

Supplement: Supplementary file 1 [file res-127-928-s001.jpg]
