# Supplementary material for: Hematoma Resolution In Vivo Is Directed by Activating Transcription Factor 1
Source: Circ Res. 2020 Jul 2;127(7):928–44. doi: 10.1161/CIRCRESAHA.119.315528 (PMC7478221; doi:10.1161/CIRCRESAHA.119.315528)

Supplemental Information

Mice were all on BL/6 background and were *Atf1*$^{-/-}$ or their littermate controls, from the European Mouse Mutant Archive, deposited by Gunther Schütz, Deutsche Zentrum fur KerbsForschung, *Prkab1*$^{-/-}$ (KOMP) (knockout first, conditional possible) or their littermate controls (http://www.informatics.jax.org/allele/MGI:4362414). The *Prkab1*$^{-/-}$ were crossed with *Frt* mice to remove the stop codon, then with BL/6 to remove the *Frt*, and then with LysMCre on BL/6 to cause conditional removal of the Prkab1 in lineages expressing Lysozyme. Genotyping of progeny *Atf1*$^{-/-}$ and *Prkab1*$^{-/-}$ was carried out by genomic qPCR for the beta-Gal insert marker allele and for the segment deleted by the recombination.

Mice were maintained on chow in a specific pathogen free facility in individually vented cages In Imperial College facilities in accordance with all institutional, national and European guidelines. All experimental mice of ages 4-6 months and of sexes 1:1 male: female. No statistically significant difference was observed with age or sex.

Anticoagulation of injected blood was as follows. A 1ml syringe containing up to 200μl concentrated citrate solution (Sigma-Aldrich S5770-50mL) was prepared with a 23G needle (depending on how much blood will be withdrawn from the mouse, citrate: blood ratio needs to be 1:5). Mice were culled by excess CO_2 plus cervical dislocation and blood immediately withdrawn using the syringe with the needle inserted into right ventricle or vena cava. The anticoagulated blood was transferred into a 1.5ml eppendorf-style tubes (VWR 211-0015) and kept on ice.

Citrate was selected as the anticoagulant because it works by chelating calcium, and clotting factor activation is calcium dependent, so anticoagulation is inherently reversible. The ratio of citrate:blood was selected as this stoichiometrically chelates plasma calcium, without excess citrate. Thus, on injection into a tissue compartment,

interstitial free calcium readily reverses the anticoagulation. This method minimises the effects of the anticoagulant, and would be ‘cleaner’ than heparin, for example.

In preliminary experiments, anticoagulated blood was compared with non-anticoagulated blood (Online Figure IIB). This indicated that anti-coagulation delayed clearance, but nevertheless allowed clearance to occur. Mouse blood coagulates *ex vivo* extremely quickly (seconds to approximately 1 minute). This made the hematoma injections (below) extremely difficult to accomplish with the necessary speed and accuracy and would therefore make reproducibility poor. Primarily to maintain reproducibility, and secondarily to allow lengthy labelling reactions (below), the reversible anticoagulation method with citrate was selected.

Fluorescence labeling of injected erythrocytes was as follows. A 1ml syringe containing up to 200μl concentrated citrate solution was prepared with a 23G needle (depending on how much blood will be withdrawn from the mouse, citrate: blood ratio needs to be 1:5). Mice were culled by excess CO_2 plus cervical dislocation and blood immediately withdrawn using the syringe with the needle inserted into right ventricle or vena cava. The anticoagulated blood was transferred into a 1.5ml eppendorf-style tubes and kept on ice. In sterile conditions, BODIPY-PC (1:100, ThermoFisher Invitrogen Molecular Probes N1148), NBD-cholesterol (1:200, ThermoFisher Invitrogen Molecular Probes D3793) or RuPIXX (1:100, preparation below) are added at the appropriate dilutions indicated to label the cells. Cell Tracker Green was used in control experiments (Thermo Fisher Invitrogen Molecular Probes C2102). The Eppendorf tube is covered with foil and placed on a rotary mixer at low speed overnight at 4°C (Stuart SB1 or Stuart SB3).

Hematomas were injected as follows. The labelled blood was kept in the dark and on ice at all times. The anesthetic apparatus is filled with the appropriate amount of isoflurane before starting and the correct sized nose cone for mice. The warming box is heated up to 35°C. The switch arrows on the anesthetic and scavenging machines are pointing towards the tubes connected to the induction chamber. The surgical area was prepared with sterile drapes, swabs and cotton buds and sterile gloves were worn. A 1ml syringe (BD 303172) with a 25G needle was prepared containing labelled blood. The mouse was placed into the induction chamber, the anesthetic machine was turned up to 4% isoflurane and 2% oxygen outflow. Once the mouse was fully sedated (breathing will be very slow), the isoflurane outflow was turned down to 2%, then the switch arrows turned so that they point towards the tubes connected to the nose cone, then mouse taken out of the chamber and placed the mouse on the sterile drape, abdomen facing upwards and its nose firmly inside the nose cone. This step was done as quickly as possible.

The mouse's right leg was held with the left hand to open up the area where the femoral artery lies. The needle was inserted gently into the skin, parallel to the mouse's leg, so that needle point is facing towards the operator. The needle point was always just below the skin so that the injection was subcutaneous. The syringe plunger is pushed so that 50µl of blood is injected subcutaneously into the femoral region, and the needle gently withdrawn out of the mouse. Any blood that may come out of the injection site is dabbed with a sterile cotton bud. The isoflurane is quickly turned off, the mouse taken out of the nose cone, weighed and put it into the warming box until it recovers from the anesthetic. Once the mouse has recovered, it is put it back into its cage.

The mouse is then left for either 8 (pre-resolution of hematoma) or 9 days (post-resolution of hematoma) before culling.

Bone marrow transplantation The entire facility is specific pathogen free and uses individual vented cages. At 12 weeks age, mice were transferred to a section of the unit with irradiator and individually vented cages, with irradiated bedding, irradiated food, acidified water containing Baytril (Bayer AG, Germany), 1 week prior to myeloablation. The mice were subjected to 4Gy + 4Gy fractionated gamma irradiation 24h apart and promptly injected intravenously with bone marrow from *Atf1*$^{-/-}$ or *Atf1*$^{+/+}$ littermate controls. Mice were maintained on irradiated food, acidified water containing Baytril for a further 2 weeks. Then 4 weeks after reconstitution, the hematoma experiment was carried as before.

Collecting the tissues Mice were culled by excess CO_2 (BOC) and cervical dislocation. Then blood was withdrawn using the syringe with the needle inserted into right ventricle or vena cava. The abdomen was dissected and tissues such as liver, spleen collected as desired. Then the lower abdomen was opened by cutting the skin down the centre of the abdomen and then the tissue to the side around the site of the hematoma and all of the tissue surrounding the leg. Then the patch of tissue containing the hematoma is excised for analysis. The excised tissue is cut in half, and one half put in a 7ml bijou containing OCT (ThermoFisher LAMB/OCT) for cryosectioning, and the other half in another bijou, containing 4% formaldehyde for fixation (VWR 9713.1000). Place the bijous are placed containing OCT on a rotary mixer at low speed overnight at 4°C. The bijous containing formaldehyde () are left at room temperature overnight. The next day, the OCT immersed tissues are frozen in in cryomoulds for cryosectioning. The tissues are oriented to enable transverse sections of the tissue to be obtained. The tissues were cryosectioned on a Bright

Cryotome (OTF-5000) at 5μm section thickness. The formaldehyde-fixed tissues are transferred into 70% ethanol and stored in the fridge until they are embedded in paraffin and sectioned at 5μm.

Validation of Ru as a fluorescent tracker of Fe

First, Ru-associated fluorescence was characterized to define whether it could be a realistic tracer of Fe (Online Figures IV-VI). Adding equimolar Ru^{3+} (Sigma-Aldrich) at a 1:100 dilution into a solution of protoporphyrin-IX (PPIX, 258385-250MG Sigma-Aldrich) in DMSO (D2650 Sigma-Aldrich) at 15°C with gentle mixing (30mins to overnight, Online Figure IVA), allowed formation of RuPPIX complexes with distinct absorption spectra and fluorescence emission (Online Figure IVB). UV-Vis Spectroscopy was on two instruments that agreed with each other: Agilent Technologies Cary 60 Spectrophotometer operating with WinUV software and Biotek Synergy HT. In Online Figure IVB, metalation by Ru produced a prominent main Soret absorption band (absorption max at 386nm) and modified the smaller Q-bands, consistent with effective Ru-N coordinate binding. In Online Figure IVC, the RuPPIX complex had a stronger primary excitation maximum consistent with emergence of stronger fluorescence. There was also more prominent excitation related to Q-bands. In Online Figure IVD, the RuPPIX complex had a brighter emission peak, with additional secondary peaks. In some preparations, this was also green-shifted. Additionally, the RuPPIX was brighter than the PPIX, so the emission spectra were recorded at a lower RuPPIX concentration to facilitate comparison. This would have the effect of minimising the observed fluorescence effect. Fourier-Transformation Infra-Red Spectroscopy (FT-IR-Spec, IR-Spec) identified a *de novo* peak in the complex at 700 cm^{-1}, consistent with successful formation of Ru-N coordinate bonds. Liquid chromatography and mass spectroscopy (LC-MS) showed

a series of *de novo* peaks in the RuPPIX complex that were not found in PPIX, further evidence of successful complexing (not shown). The (lipophilic) RuPPIX was then incorporated into erythrocytes by simple co-incubation allowing passive diffusion at 4C overnight on a rotator, and brief washing by pelleting at 13,000g 60s in a Sigma refrigerated benchfuge () and resuspension in PBS.

Macrophages that had been incubated with RuPPIX developed green fluorescence emission that was distinct to RuPPIX itself and corresponded to fluorescence emission with Ru-oligo-His complexes or Ru-ferritin complexes (Online Figure VA). Metals coordinate in ferritin to N-atoms of His residues. Green fluorescent emission was measured from complexes of Ru with Ferritin in a cell-free system (Online Figure VB) (horse spleen apoferritin, Sigma-Aldrich A3660). This fluorescence corresponded to the green fluorescence from Ru-oligo-His complexes (Online Figure VC) (Oligo-His, Ab14943). By confocal analysis, emission spectroscopy from macrophages that were incubated with Ru-PPIX for 48h was also consistent with the spectral characteristics of the Ru-oligo-His and Ru-Ferritin complexes (Online Figure VD). In each of Online Figures VB-D, fluorescence emission from Ru-complexes were higher than the equivalent Fe controls, which had no significant emission above background. Whilst heme (Fe-PPIX) induced HO-1 mRNA (*Hmox1*, Figure 5E) and protein (Figure 5F), Ru-PPIX did not induce HO-1 protein, weakly induced mRNA on its own, and had no additive effect on Hmox1 mRNA induced by heme (Figure 5 E-F).

The cell fluorescence is response to Ru-PPX was not seen with heme, and was suppressed by si-RNA to *Hmox1* (not shown). Since HO-1 catalyzes conversion of Fe-PPIX to Fe that rapidly trafficked into Fe-Ferritin complex, this is consistent with

Ru tracking Fe. Cell lysis (in proprietary nuclear lysis buffer, buffer AM-1 Active Motif 70020367) and centrifugation revealed insoluble deposits of pigment in the RuPPIX-treated cells. Native (non-denaturing) PAGE of these deposits showed a dominant band at very high molecular weight (>200kDa) (not shown). LC-MS of these deposits showed that adding Ru caused conversion of a peak corresponding to the FeO core of ferritin-Fe, into a higher mass material, consistent with incorporation of the heavier Ru (not shown).

Bone marrow macrophage culture

Legs were removed from culled mouse, the femur was separated from tibia and fibula, and the ends of the femur were cut using a no. 20 size scalpel. Then, bone marrow was flushed out of each femur and tibia using a 25G needle on a 1ml syringe containing DMEM media (21909-035) with 10% FCS and 10% L929-conditioned media. The flushed bone marrow (in approximately 4ml media) was made up to 50ml of DMEM media + 10% FCS + 10% L929 media. The 50ml media containing bone marrow cells was divided into 4 T75 flasks and cultured at 37ºC, 5% CO_2 for 6 days. Hemin was from Sigma-Aldrich (Fluka 51280) and was aseptically prepared in DMSO (D2650) at 25mM and sterile-filtered and endotoxin-tested.

QPCR

All QPCR was carried on on a BioRad CFX96 instrument. Stock primers and cDNA were diluted by 1:10 (5ul primers/cDNA added to 45ul molecular biology water). Master-mix was made up for the appropriate number of wells, with containing the following:

MESA Green 7.5ul

Forward Primer 0.25ul

Reverse Primer 0.25ul

Water 4.0ul

Total 12ul per well

2ul of diluted cDNA added to each well. Each sample was run in triplicate.

The qPCR protocol was as follows:

1. 95ºC for 3 mins
2. 95ºC for 10 secs
3. 60ºC for 1 min
4. Repeat (Steps 2-3) 40 times
5. Plate read melt curve 56ºC to 95ºC for 5 secs

For primers producing a product longer than 150bp, the following protocol was used:

1. 95ºC for 3 mins
2. 95ºC for 10 secs
3. 60ºC for 2 mins
4. Repeat (Steps 2-3) 40 times
5. Plate read melt curve 56ºC to 95ºC for 5 secs

With primers for human genes, the protocol was as above, but carried out at 60°C extension temperature. Mesa Green is a SyBr Green mastermix with ROX, (Eurogentec, RT-SY2X-03+WOUFL). Primers were custom-ordered from Eurofins (Supplemental Table).

Histology and immunohistology.

Cryosections and paraffin sections were cut at 5μm thickness. Cryosections and deparaffinized sections of the hematomas were stained with H&E (Hematoxylin VWR 350604T, Eosin Merck 1.09844.1000), Oil Red O and Mayer's haematoxylin, Berlin Blue (Perls', Prussian Blue) stain for iron, or immunolabeled, or imaged for the fluorescent tracker, as indicated. Sections were analyzed blind. Immunohistochemistry was performed by standard procedures on residual sections not required for analysis of lesion size. Primary antibodies, which were diluted as appropriate in PBS, were rat anti-CD68 (both from Serotec, Oxford, UK, MCA1957A488), alkaline phosphatase conjugated mouse anti-alpha actin (clone α1A4) (Sigma-Aldrich, Poole, UK). Primary antibodies were followed by biotinylated rabbit or goat anti-rat immunoglobulin (Ig) secondary (Dako E0432) and ABC-peroxidase system (Dako) using 3,3'-diaminobenzidine tetrahydrochloride (DAB) as substrate (Vector Labs SK-4100).

Immunolabeling – peroxidase

Slides were air dried for ½ hr at room temperature (RT, 15°C), then fixed in acetone for 5 min on ice. Then, slides were air dried for ½ min, and not allowed to dry after this step. Slides were then washed in PBS 2 x 4 min (PBS used throughout labeling is Ca- and Mg- free). The sections were next blocked with 10% normal serum in PBS for 60 mins at RT. Normal serum is the serum of the species that the secondary

antibody was raised in, and is used heat-inactivated. Then the sections were washed in PBS for 5 mins, and then blocked with Avidin (Vector Laboratories, Peterborough, UK Vector Blocking Kit- Catalog No. SP2001) undiluted for 15mins at RT in a moist chamber, and then washed again in PBS for 5 mins. Then the sections were blocked with biotin solution (Vector Blocking kit Catalog No. SP2001) for 15 mins RT in a moist chamber, and again washed in PBS for 5 mins.

Primary antibodies were as follows:

Spectrin (Abcam Ab11751), p-ATF1 (Abcam 76085), HO-1 (Abcam Ab13243), Cd68 (Serotec MCA1957), NFκB (Abcam Ab32536), NOS2 (Abcam Ab110282), 3-nitro-tyrosine (Abcam Ab110282), 8-oxo-dG (Trevige 4354-MC-050), Perilipin-2 (Novus NB110-40877),

Control antibodies were as follows: IgG1κ (Abcam Ab170190), rabbit monoclonal IgG (Abcam Ab172730), rat IgG2a (Serotec MCA1212), mouse IgG2b (Abcam Ab170192), rabbit IgG (Novus Nbp2-42891). Appropriate IgG controls were used throughout.

Secondary antibodies were as follows: rabbit anti-rat IgG (Vector BA-4001); goat anti-rabbit IgG (Vector BA-1000); AlexaFluor tyramide 488 kit (ThermoFisher BA40932).

Next, primary antibody was added, and incubated for 45 mins at RT in a moist chamber at 0.5-10μg.mL^{-1} in PBS with 5% normal serum, concentration depending on the clone and optimisation. Controls were rabbit monoclonal IgG or isotype controls as in the Slides were then washed in PBS for 5 mins at RT, and then incubated with biotinylated secondary antibody at 1:400 in PBS, for 40mins at RT in

a moist chamber. Next, the ABC complexes were prepared, using the Vector ABC-Elite-Standard Kit for Peroxidase (Vector Catalog. No. PK6100). To 100μL of PBS was added 1μL solution A (Avidin) from the kit, mixing well. Then 1μL of solution B (Biotin-Peroxidase) is added from the kit, mixing well. The mix was allowed to stand for 30 min for the complexes to form and was then ready to be used.

At the end of both these incubations, the slides are washed in PBS for 5 mins, and endogenous peroxidase inactivated by incubating sections for 15mins in 0.3% hydrogen peroxide in PBS at RT. Slides are washed again in PBS for 5 mins, and then incubated with the prepared ABC complexes for 40 min at RT in a moist chamber.

DAB solution was prepared as follows: to 5ml distilled water, add 2 drops buffer, 4 drops DAB (Vector Laboratories SK-4100), 2 drops H_2O_2. Slides were then washed in PBS for 5 mins, and then incubated in DAB solution for 3-10 min (it helps to place a sheet of white paper under the tray to observe the color change). The reaction was stopped by washing in PBS for 5 mins. Slides were then counterstained with Haematoxylin (VWR 350604T) for 30 sec, then wash with tap water until the water runs clear, then washed in Scott's water tap substitute 5 min (If required, sodium bicarbonate Sigma-Aldrich S6014-500G), then dehydrated in 70%, 100% ethanol (1 min each) and coverslip, using permanent mounting medium (e.g. Histomount, National Diagnostics HS-103). Slides were dried horizontally overnight before racking.

Vector Red Immunolabeling

Frozen sections were cut at 5μm thickness. Frozen sections were air dried for 30 mins, fixed in ice cold acetone for 5 mins, washed in 1x PBS 5 mins, blocked in 10% serum (species secondary antibody raised in) for 1 hour. Then, excess serum was tapped off and slides are washed in PBS briefly. Sections were incubated with Avidin blocking buffer for 15 mins, washed in PBS briefly, then incubated with Biotin blocking buffer for 15 mins, and then briefly washed in PBS.

The sections were next incubated with primary antibody for 45 mins, the excess was tapped off and sections washed in PBS for 5 mins. Next, sections were incubated with biotinylated secondary antibody (1:100) for 20 mins and washed briefly in PBS. Then, Avidin Alkaline phosphatase (1:100) was added for 20 mins, slides are briefly washed in PBS. Next, TBS pH9 was added for 1 min (prepared in-house, 25mM Tris 150mM NaCl (TrisHCl, T3253-500G, Tris base T1503-1KG, NaCl S9625-1KG, all Sigma-Aldrich).

Fresh Vector Red solution was made up as follows: to 5ml 100mM Tris-HCl pH 8.2-8.5 add 2 drops of reagent 1, 2 drops of reagent 2 and 2 drops of reagent 3 from the Vector Red alkaline phosphatase substrate kit (Vector Labs, SK-5100). Sections were incubated in Vector Red solution for 20 mins, washed in PBS 5 mins, incubated in DAPI (1:500) solution for 10 mins, washed briefly in PBS and mounted in Vectashield (Vector Laboratories PK-4000).

Immunofluorescence labeling of deparaffinized hematoma sections

Paraffin sections were cut at 5μm thickness. Paraffin sections were dewaxed in Histoclear for 5 mins, dehydrated in 100% the 70% ethanol for 5 mins each, then rehydrated in distilled water for 5 mins. Antigen retrieval was performed by heating in

a microwave at 60% power for 10 mins, with slides immersed in sodium citrate solution. After cooling for 5 mins, the slides were incubated in 0.03% hydrogen peroxide for 15 mins, washed briefly in 1x PBS, blocked in 1% BSA in PBS (A2153-50G, Sigma-Aldrich in Invitrogen 20012-019) for 1 hour. Then, excess BSA was tapped off and slides are washed in PBS briefly. The sections were next incubated with primary antibody diluted in 1% BSA for 1 hour, the excess was tapped off and sections washed in PBS for 5 mins. Next, sections were incubated with biotinylated secondary antibody (1:100) for 40 mins and washed in PBS for 5 mins. Then, sections were incubated with Streptavidin-HRP diluted by 1:100 in 1% BSA for 1 hour and washed in PPBS for 5 mins. Following this, the sections were incubated in Tyramide working solution (Tyramide kit, Invitrogen T20932, (ThermoFisher); Tyramide Alexa Fluor 488 diluted by 1:100 in amplification buffer, Invitrogen,) for 10 mins, washed in PBS for 5 mins, incubated with DAPI nuclear stain (1:500 in PBS) for 10 mins before washing in PBS for 5 mins and mounting a Vectashield (Vector Laboratories) prior to imaging.

Morphometry and hematoma quantification

The haematoma boundary of each subject's tissue was identified based on the observation of erythrocytes visible by H&E staining, and Spectrin-positive immunofluorescence. To obtain the number of cells per mm^2 within the hematoma in H&E-stained sections, haematoxylin stained nuclei were counted using the counting tool in ImageJ (https://imagej.nih.gov/ij/). The total area of the hematoma was also measured using the polygon tool in ImageJ and calibrating the software using the scale bar produced by the Leica imaging software associated with the light

microscope. The total cell count was divided by the hematoma area to obtain the cell density.

The Spectrin-positive percentage area of the hematoma was obtained by outlining the hematoma area using the polygon tool in ImageJ and calibrating the software to identify green fluorescence in the outlined area. The positively stained area was expressed as a percentage of the total area.

Immunolabeling was quantified by the most appropriate one of a number of methods. Cell lineage markers were expressed as % positive in lesional cells, relative to total cell counts based on a nuclear dye.

Cell signaling molecules or induced genes were quantified by an adaptation of western blot quantification, in Image J. In this, a region of interest was drawn around the hematoma, and an image histogram exported. This was used to define immunolabeling extent. Background signal was measured on the isotype or IgG-only control. The simpler measurement was % lesion area that was clearly above background (immunolabeling threshold). This is simple threshold-based planimetry.

A more complex but related calculation was the integral or area under the curve. With this, pixel intensity is multiplied by the number of pixels of that intensity, and the product is summed. This (in principle) provides a more accurate and more robust measure of the total amount of protein in a section. Simple planimetry (% area above threshold) and area x intensity correlated extremely well, and the data shown are only % area, as this was the simpler and clearer variable. Thus, morphometry was by threshold-based planimetry.

Macrophage culture and stimulation

Macrophage culture from humans and from mice exactly according to our previous methods.

Human peripheral blood was obtained with informed consent and institutional ethical approval. PBMCs were prepared by density-gradient centrifugation and monocytes purified by adhesion as before. Human aseptically prepared LDL was purchased from Calbiochem (437644-10MG) and oxidatively modified with hypochlorite 1mM exactly as in our previous papers.

Bones were collected aseptically from ATF1-KO, AMPK-KO or matched littermate control mice and flushed with PBS to collect bone marrow cells. The cells were then cultured in six T75 flasks in 10% FCS DMEM supplemented with antibiotics, L-Glu and 10% L929-conditioned medium (a source of M-CSF) for 7 days. Cells were then washed and adherent cells recovered by scraping into medium, counted and transferred to fresh 10% FCS DMEM in 24-well plates or 96-well plates. RT-qPCR Macrophages were lyzed in a guanidinium-based buffer (RLT buffer, RNA-Easy Mini kit, 74104, Qiagen Manchester, UK) and RNA purified by silica-resin affinity (according to manufacturer's instructions). Reverse transcription was with Invitrogen Superscript-II, following manufacturer's instructions (Invitrogen, Life Sciences, Paisley, UK, 18064022). Quantitative polymerase chain reaction (qPCR) was with ausing a BioRad iCycler CFX96 real time PCR, MesaGreen mastermix (Sybr Green/Taq, Eurogentec, Southampton, UK, RT-SY2X-03+WOUFL) and 100fmol.μL^{-1} custom primers (synthesized by MWG Biotechnology, Ebersberg, Germany). Primers were designed by a Primer3 algorithm accessed via the NCBI website (www.ncbi.nlm.gov). Additional validation was on a further five donors over a longer

time course, using qPCR by the same methods. Primer sequences are in the Supplemental Table.

Gene knockdown

siRNA was carried out according to our previous methods. The only modification was that mouse-targeting sequences were purchased rather than human-targeting sequences. The si-RNA oligos were from Dharmacon and were pools of 4 sequences and were controlled for with non-targeting si-RNA from the ame manufacturer at the same concentration and prepared in the same way at same time. All si-RNA were stored at -80°C. Si-RNA was made into complexes with Interferin (PolyPlus, Strasbourg, France, 409-10) by incubation of 100 pmol siRNA in 1μL with 1μL Interferin for 5-10 min. The complexes were thendiluted in 100μL medium and added to a well ofcells in 1 mL medium with 10% serum for 24 hours, at 100pmol siRNA per 10^5 cells in each well. The si-RNA complexes were incubated with the macrophages for 18-24h before stimulation.. Where multiple wells were treated, the incubation volumes of the mix were scaled by direct proportion.

Microarray analysis was carried by a minor adaptation of previous methods

Macrophages were lyzed in guanidinium (RLT buffer, Qiagen, Crawley, UK) and RNA purified by silica-resin affinity (RNAEasy Mini, Qiagen, according to manufacturer's instructions). Sample quality was controlled with Nanodrop UV spectrophotometry and Bioanalyzer micro-LC. Samples were prepared for Agilent 4x44k and Affymetrix 1.0ST microarrays following respective manufacturer's instructions. The service Oxford Genome Technology (OGT) was used for the array labelling, hybridisation and reading (in contrast to before, which was with collaboration with UniversitätsSpital Zürich). A dual-colour system was used as

before, but run against reference rather than treated and control. The microarray glass slides were also Agilent 2-colour, but using mouse probes rather than human and an updated denser matrix with improved annotation. The same RNA samples were used for both microarray platforms and both analyzed with GeneSpring GX12 Agilent. Initial validation was with quantitative polymerase chain reaction (qPCR) on the same RNA samples after reverse transcription (Invitrogen Superscript, manufacturer's instructions) using BioRad iCycler real time PCR, MesaGreen mastermix (Sybr Green/Taq, Eurogentec, Southampton, UK) and 100fmol.μL -1 custom primers (synthesized by MWG Biotechnology, Ebersberg, Germany). Primers were designed by Primer3 (www.ncbi.nlm.gov). Additional validation was on a further five donors over a longer time course, using qPCR by the same methods. Bioinformatics Gene Ontology analysis was with GeneSpringDAVID and PANTHER, and manual curation of GO-terms. Transcription factor (TF) binding site analysis was primarily with LASAGNA2.0 and PASTAA.

Experimental Details

Sample preparation

Stock solutions of PPIX (Sigma-Aldrich 258385-250MG), RuPPIX and $RuCl_3$ (10 mM, Sigma-Aldrich 206229-1G) in DMSO were stored at -80°C and thawed directly before use. All samples were diluted a thousand-fold to 10 µM in PBS buffer (ThermoFisher, tissue culture grade, 10010023) for the photophysical measurements.

Absorption spectroscopy

UV-visible absorption spectra were measured using an Agilent Technologies Cary 60 Spectrophotometer operating with WinUV software. The sample was held in a quartz cuvette with a path length of 1 cm. Absorption spectra were recorded against a baseline of pure solvent in an optically matched cuvette with a scan rate of 600 nm / min and a data interval of 1.0 nm.

Fluorescence Spectroscopy

Emission and excitation spectra were acquired on an Agilent Technologies Cary Eclipse Fluorescence Spectrophotometer, in quartz cuvettes with a path length of 1 cm. Emission and excitation spectra were collected with a scan rate of 120.0 nm / min, a data interval of 1.0 nm and band-passes of 5 nm. Excitation (λ_{ex}) and emission wavelengths (λ_{em}) for PPIX, RuPPIX and $RuCl_3$ were 390 nm and 615 nm respectively.

ELISA

ELISAs for LTB_4, RvD_1 and PGI_2 were carried out on saved culture supernatants. Cayman Chemicals 96-well colorimetric ELISA kits were used, exactly according to manufacturer’s instructions (CAY520111, CAY500380, CAY50110). The same kit was used for both species (human and murine eicosanoids are chemically identical). Absorbance was measured at the manufacturer’s recommended wavelength, using a Tecan SpectraFluor 96-well plate reader. ELISAs for serum Amyloid A (SAA) were purchased as the DuoSet kit for mouse SAA (R&D Systems DY2948-05) and were used as per manufacturer’s instructions, including the calibration in duplicate and samples in technical triplicate.

Western blotting

RAW cells were lysed in Laemmli buffer (10%SDS (Sigma-Aldrich 71725), 20% glycerol (Sigma-Aldrich G5516, TrisHCl-pH6.8, 1:100 phosphatase inhibitors (Active Motif phosphatase inhibitor cocktail 37493) no bromophenol blue or 2-ME)) and subjected to SDS-PAGE with a Novex system (Invitrogen kit NP030A)(Invitrogen NuPAGE precast BisTris 4-12% gradient (NP0321BOX), MOPS running buffer NP0001) and Rainbow Markers RPN800E, electrotransferred using Novex to Immobilon-FL (ThermoFisher Pierce 88518). Membranes were blocked for 10mins with 10% BSA TBS and incubated overnight with 1:1000 anti-HO-1 (rabbit monoclonal Abcam Ab52947) or anti-beta-actin (Abcam Ab6276) and then developed respectively with anti-rabbit or anti-mouse Peroxidase-labelled (Dako P0447 and P0448) and ECL-Plus (ThermoFisher Pierce 32132) and Amersham Hyperfilm (VWR 28-9068-37) and exposed over a log range, and the exposure with highest signal:noise ratio used.

Spleen Berlin Blue staining and evaluation

Formalin-fixed paraffin embedded sections of spleen were dewaxed in limonene, taken through graded alcohols to water. Sections were then stained for Berlin Blue (Prussian Blue, Potassium ferrocyanide complex reaction) a classical method for iron staining. The Berlin Blue was a freshly prepared 1:1 mix of 2% HCl (H1758-100mL) and saturated potassium ferrocyanide (Sigma-Aldrich P8131) for 20 minutes. Counterstaining was with 0.4% Neutral Red in water 10 minutes (Sigma-Aldrich N6634). Sections were immediately imaged with RGB camera and brightfield microscopy (Leica DM2500). All spleens for this analysis were batch-processed for

consistency and equivalence of timing and conditions. Images for morphometry were taken at x4 and under identical settings.

The Berlin Blue / ORO dual-stained sections, cryosections were first stained with ORO and then with Berlin Blue according to standard protocols as follows:

Oil Red-O - Perls Staining Protocol

Reagents

Oil Red-O powder (Sigma O0625-25G)
Isopropanol (VWR 20922.320)
Dextrin powder (Sigma D2006)
4% paraformaldehyde (VWR 28794.295)
Vectashield (Vector labs H-1000)
Cover glasses (VWR 631-0880)
Potassium ferrocyanide (Sigma-Aldrich P9387)
Hydrochloric acid (Sigma-Aldrich H11758)

Making Perls’ working solution

Stock solution A
Potassium ferrocyanide 2g
Distilled Water 100ml

Stock solution B
Hydrochloric acid 2ml
Distilled Water 100ml

Working solution
Stock solutions A and B mixed at 1:1 ratio immediately before use in the fume hood.

Make Oil Red-O working solution

1. Dissolve 2g of Oil Red O powder in 200ml of isopropanol in a fume hood.
2. Dissolve 1.2g of Dextrin powder in 120ml of dH20.
3. Swirl both solutions in their 500ml Schott glass bottles and leave overnight to dissolve at room temperature.
4. Filter the Oil Red-O solution to remove any residual powder.
5. Combine with the Dextrin solution to make the Oil Red-O working solution.

<u>Staining protocol</u>

1. Air dry the slides to be stained for 30 minutes.
2. Melt the Kaiser's glycergel at 50°C in a water bath.
3. Fix in sections in 4% paraformaldehyde for 10 minutes.
4. Wash in distilled water for 5 minutes.
5. Wash in 60% isopropanol for 1 minute.
6. Immerse the slides in Oil Red-O working solution for 15 minutes.
7. Wash off the excess Oil Red-O solution in distilled water with agitation.
8. Place in working solution for 24 hours in fume hood.
9. Rinse with distilled water and agitation as necessary.
10. Mount with aqueous Vectashield mounting medium and cover glass.

Berlin Blue morphometry was as follows in Image J.: RGB Split, select Red channel (absorbs blue). Check red and blue channels look like white and red pulp respectively. Invert Red channel. Draw region of interest (ROI) to exclude artefact and only include spleen. Image histogram. Export to Excel. Calculate total pixels in image. Calculate pixel intensity x number at that intensity and sum up all (sum of pixel intensity x number at that intensity). This is the integral of staining intensity and staining extent, and then divide this by total pixels. This now expresses the average amount of stainable iron across the ROI. Do this for each mouse.

Statistics

Data were graphed in stem-and-leaf plots and examined visually for distribution, and tested for normality using Shapiro-Wilk when $n \geq 5$. SigmaPlot was used for routine analysis. SigmaPlot reports exact p-values down to $p<0.001$. GraphPad Prism was used for exact p-values down to $p<0.0001$. SPSS was used for remaining exact p-values. SPSS was used for time-series analysis.

Animals were randomized using the randomization function in Microsoft Excel (=RAND()). Alternate allocations were made for values over or under 0.5.

Power calculations for hematomas were made on the basis of our own preliminary data indicating that hematomas were uniformly present at day 8 and uniformly absent at day 9 in normal mice. Using a two-group non-parametric assessment, n=4 in each group would be sufficient. However, it was possible that individual variables assessing mechanism would have a high variance or be non-normally distributed and it would be important to adequately power these in addition. Therefore, preliminary power calculations were based on Power = 0.85, alpha=0.05, an expected difference of 20% of mean, and a standard deviation of 10% of mean (ie CV=10%). This yields a target n-value of n=8. For *in vitro* measurements of gene expression, power calculations were based on normality, Power = 0.85, alpha=0.05, expected difference of 50% of mean, and a standard deviation of 20% of mean (*i.e.* CV=10%). This suggests a target n-value of n=5. Rounding to numbers of mice within each cage in practice caused any deviations from these targets.

Data that passed normality by Shapiro-Wilk, visual inspection, general acceptance of normal distribution, and n≥5, were tested as follows. Inherently paired data were tested using Student's paired t-test. Non-paired data in two groups were tested using Student's t-test. Data in several groups, were tested using One Way ANOVA and post-tested using Holm-Sidak correction for multiple simultaneous comparisons. Time-series data were significance tested using Repeated Measures One Way ANOVA.

Data that failed normality testing were significance tested as follows. Data in multiple groups were tested using One Way ANOVA on Ranks (Kruskal-Wallis), with Dunn's

post-test. Unpaired data in 2 groups were tested using Wilcoxon Rank Sum Test (Wilcoxon-Mann-Whitney).

No corrections for multiple testing were made across tests (*i.e.* only within-test corrections were made), When any comparison is not shown it was not run. In several figures, only 1 comparison between 2 treatments was key (*e.g.* Day 9, KO vs control) and this comparison was chosen in advance. Whenever a multiple comparison procedure is referenced, a post-hoc p-value correction was used

Online Figure Legends

Online Figure I. Myeloid AMPK and hematopoietic ATF1 are required for erythrocyte clearance at 9 days

Scoring of hematomas in mice bone marrow transplanted The bone marrow transplantation from *Atf1*$^{-/-}$ or *Atf1*$^{+/+}$ littermate control mice into *Atf1*$^{+/+}$ recipients was carried as in Supplemental Methods. The difference between experimental groups was so profound that hematomas were simply scored as present or absent. (The experiment overlapped the start of the international COVID lockdown, and the research histology facility closed so tissue processing and sectioning was not an option). Exact p-value given, Mann-Whitney.

Online Figure II Preliminary experiments for hematoma model

A, Time course of hematomas Cryosections are shown for a series of time points as indicated. The control image is the contralateral side for the day 2 image. On the basis of these, days 8 and 9 were selected for an experiment with more mice and paraffin sections were chosen for improved resolution. SkM – skeletal muscle, Fasc – fascia, Hem – hematoma, Epi- epidermis. Scalebars, 100µm.

B, Comparison of anti-coagulated and intact blood. Representative images, timepoint and addition of citrate anticoagulant are shown. H – hematoma (or its residue), Epi – epidermis, SkM – skeletal muscle. Scalebars, 100µm.

C, Hematoma injection does not influence Serum Amyloid A at 48h. In the mouse, serum amyloid A (SAA) is the equivalent of human C-Reactive Protein (CRP)as an acute phase reactant. They are both homologues and induced in the liver rise in response to inflammatory cytokines. In mouse, subcutaneous TNF-α

induces SAA at 48h, so this time-point was chosen. Exact P-value as indicated, Mann-Whitney. Hematoma injection or saline control as indicated. SAA was measured by ELISA in pg.mL^{-1}.

D, Prolonged hematoma in *Atf1*$^{-/-}$ mice. Images are H&E, respectively x10 and x20, representative of different features (n=3). There was essentially almost complete resolution with no real pathology. Epi – epidermis, SkM – skeletal muscle. Brown arrowheads – hemosiderin-laden macrophages. Scalebars – indicated distances.

Online Figure III Aspects of phenotype of *Atf1*$^{-/-}$ mice

A, haematocrit. Blood from 12 week old mice was clotted and separated into red cells and the components weighed. Haematocrit was calculated from the red cell mass divided by the total mass. Groups are as indicated Exact P-value as indicated, Student's t-test (data passed Shapiro-Wilk and visual inspection and variable is widely accepted to be normally-distributed).

B, spleen mass. 12 week old mice were culled and weighed, the spleens were weighed and spleen mass expressed as a % of total body mass. *Exact P-Value, Student's t-test (data passed Shapiro-Wilk and visual inspection and are widely accepted to be normally-distributed).

C, *Atf1*$^{+/+}$ Splenic red pulp HO-1+CD68+ macrophages. Spleen were cryosectioned and stained with HO-1 / CD68 immunostaining (Methods), DAPI counterstained and imaged with an RGB camera, true colours, stains as indicated. Foll - lymphoid follicle (white pulp) RP – red pulp. Scalebars as indicated. Images representative of n=4 spleens. No staining seen in negative controls at these camera settings.

D, *Atf1*$^{-/-}$ Splenic red pulp HO-1+CD68+ macrophages. Spleen were cryosectioned and stained with HO-1 / CD68 immunostaining (Methods), DAPI counterstained and imaged with an RGB camera, true colours, stains as indicated. Foll - lymphoid follicle (white pulp) RP – red pulp. Scalebars as indicated. Images representative of n=4 spleens. No staining seen in negative controls at these camera settings.

E, *Atf1*$^{-/-}$ Splenic iron (in splenic red pulp macrophages). Spleen iron is normally located in splenic red pulp macrophages. Spleens from *Atf1*$^{-/-}$ and *Atf*$^{+/+}$ littermate control mice were sectioned and stained with Berlin Blue (Perls, Prussian Blue,

ferrocyanide) and counterstained with neutral red by standard protocols. Foll - lymphoid follicle (white pulp) RP – red pulp. No obvious difference was observed between *Atf1*$^{-/-}$ and *Atf*$^{+/+}$. Representative n= 7 (*Atf1*$^{-/-}$) and n=9 (*Atf*$^{+/+}$). That is, there was an unremarkable location and amount of splenic red pulp iron in splenic red pulp macrophages. Scalebars = 100µm.

F, Iron content of spleens by histomorphometry of Berlin Blue-stained sections. Y-axis, iron estimates (arbitrary units). X-axis, groups as indicated. Each point represents a different mouse. Exact P-value as indicated (P=0.597, Mann-Whitney). [also data passed Shapiro-Wilk and visual inspection, and would be expected to be normally-distributed, mean±SE *Atf1*$^{+/+}$ 218.4±3.5 n=9, *Atf1*$^{-/-}$ 219.6±5.0, n=7, P=0.843 Student's t-test]

Online Figure IV Generation of protoporphyrin-IX-Ruthenium complexes

A, reaction to form RuPPIX.

B, absorption spectra of RuPPIX complex and Ru and PPIX precursors (as indicated). X-axis, wavelength of incident light (nm). Y-axis, specific absorbance for each compound.

C, excitation spectra of RuPPIX complex and Ru and PPIX precursors (as indicated). X-axis, wavelength of incident (exciting) light (nm). Y-axis,corrected fluorescence emission for each compound at standard wavelength (615nm).

D, emission spectra of RuPPIX complex and Ru and PPIX precursors (as indicated). X-axis, wavelength of emitted light (nm). Y-axis, emission intensity for each compound at its own maximum excitation wavelength (RuPPIX 395nm, PPIX 381nm, Ru no detectable fluorescence, but 390nm selected). The PPIX was measured at a higher concentration than the Ru-PPIX complex (as indicated) to present them on

the same graph as the RuPPIX complex had far higher fluorescence. This only has the effect of making the RuPPIX emission seem less bright.

E-F, Fourier Transform Infrared Spectroscopy (FT-IR-Spec) spectra of the synthetic complex Ru-PPIX and precursors Ru and PPIX as controls (as indicated). Each compares Ru, PPIX and RuPPIX complex under equivalent conditions as indicated. E, Full FT-IR-spectrum of RuPPIX, PPIX and Ru for reference. There is a peak unique to the RuPPIX complex at 700 cm^{-1} in the RuPPIX trace, which is consistent with the range of literature reports documenting FT-IR of Ru-N coordinate bonds. F, Magnified view of image in E over range 650-800 cm^{-1}, more clearly showing the Ru-PPIX-specific peak at 700 cm^{-1} that is consistent with the Ru-N coordination bond.

G, reproducibility of RuPPIX-related fluorescence. Fluorescence measurements taken on independent instrument (Tecan Spectrafluor Plate Reader). X-axis, filter pairs as given Ex/Em wavelengths (nm), Y-axis fluorescence emission (arbitrary fluorescence units). Relative to control erythrocytes, erythrocytes incubated with Ru-PPIX exhibit specific green fluorescence, wavelengths similar to FITC. Compounds are RuPPIX complex or PPIX or Ru precursors. Data are mean±SE, n-5

H, Linearity of RuPPIX-related fluorescence. X-axis, concentration of RuPPIX, Y-axis, fluorescence (AFU). Data are mean±SE, n=5.

Online Figure V Ru-associated fluorescence in macrophages is consistent with interaction with histidine residues.

A, Fluorescence in erythrocytes. RuPPIX was incorporated into erythrocytes. RuPPIX-containing erythrocytes are visible using standard FITC filter settings (485±10nm excitation, 535±10nm emission). Green arrowheads, green-fluorescent erythrocytes. Scalebars = 100µm.

B, Fluorescence from complexes of Ru or Fe with ferritin (Equine spleen apo-ferritin, Sigma-Aldrich) at 10μM. Y-axis, specific fluorescence emission (AFU, corrected for Ru and ferritin background controls). X-axis, Excitation-max / Emission-max filtersets. Bars as indicated. Data are mean ± SE n=5.

C, Fluorescence from complexes of Ru with oligopeptides (oligo-His, oligo-Trp, Bachem) or polypeptides (polyglutamate, Sigma-Aldrich). Metal ions in ferritin coordinate to His. Y-axis, specific fluorescence emission (correcting for Ru and ferritin background controls). X-axis, Excitation-max / Emission-max filtersets.

Bars as indicated. Data are mean ± SE n=5.

D, Representative series of confocal images taken using the Meta attachment on a Zeiss LSM 780. This allows image emission spectrometry, taking a new image every 10nm of excitation wavelength. Macrophages were incubated 48h with each reagent as indicated, at 10μM. Scalebars = 100μm.

E, Fluorescence quantification of images in C. Y-axis, fluorescence intensity. X-axis, emission wavelength. Reagents are as indicated. Data are mean±SE.

F, Heme induces *Hmox1* mRNA in RAW cells. Y-axis, *Hmox1* mRNA measured by RT-qPCR and ΔΔCt method. X-axis, treatment as indicated. RuPPIX only weakly induces *Hmox1* mRNA relative to heme. Timepoints all 4h, concentration 10μM. Each point represents a biological replicate. Mean±SE are superimposed.

G, Western blots for HO-1 (rabbit monoclonal, Abcam, Immunoperoxidase, Pico West, Hyperfilm) or beta-actin loading control (Abcam). Treatments of the cells, antibody used to blot, and adjacent Mr markers are shown as indicated, timepoints all 4h. Heme induces HO-1 protein in RAW cells. RuPPIX does not induce HO-1 protein in RAW cells. Full blots with all exposures and markers and product insert are shown in an additional Supplemental File.

Online Figure VI Small macrophages in experimental hematomas express HO-1 and store metal from erythrocytes

A, Cell size of all macrophages or HO-1+ve macrophages.

Cell size was measured in macrophages positive for Cd68 (all macrophages) or for HO-1 (Mhem macrophages) (Methods). Cells positive for Cd68 or for HO-1 were drawn around in Image J and cell area measured. Each graph is a histogram of macrophages in hematoma in an individual mouse, Y-axis, number of cells, X-axis, cell size (arbitrary units). Macrophages form a bimodal distribution, and HO-1-positive macrophages comprise the smaller subpopulation.

B, Tracking of fluorescent lipids or metal into large and small macrophages. Macrophage size was measured as in Panel A. Macrophage sizes were measured in hematomas from the labeled erythrocyte experiments, as in Panel A, but focusing on macrophages containing the indicated fluorescent tracker. X-axis, label that has tracked into macrophages, Y-axis, cell size (calculated in A), n=4 per group. Exact P-Value indicated (Mann-Whitney, as this comparison was selected *a priori* as the most important comparison). Fluorescent lipids track into larger macrophages and fluorescent iron analogue (Ru) into smaller macrophages.

Online Figure VII. *Atf1* gene deletion does not impair leukocyte clearance.

Peripheral blood leukocytes (neutrophils) were prepared by Ficoll-Hypaque centrifugation and killed by exposure to UV-light. **(A)** an initial time-course indicated that the optimum measurement window was day 1-2 after initial injection.

(B) Injection of fluorescent-labelled leukocytes showed complete clearance between 1-2 days after injection in control mice and in mice deficient in *Atf1*. Green arrowhead, fluorescently-labelled leukocytes. Leukocytes were labelled with Cell Tracker Green and nuclei labelled with DAPI (blue). Scalebars = 100μm. Representative, n=4 mice each time-point and each genotype.

(C) H&E examination of tissues showed complete clearance of leukocytes between 1-2 days after injection in control mice and in mice deficient in *Atf1*. Images representative of n=5 mice. Wbc – white blood cells. Scalebars = 100μm. Representative, n=4 mice each time-point and each genotype.

Online Figure I

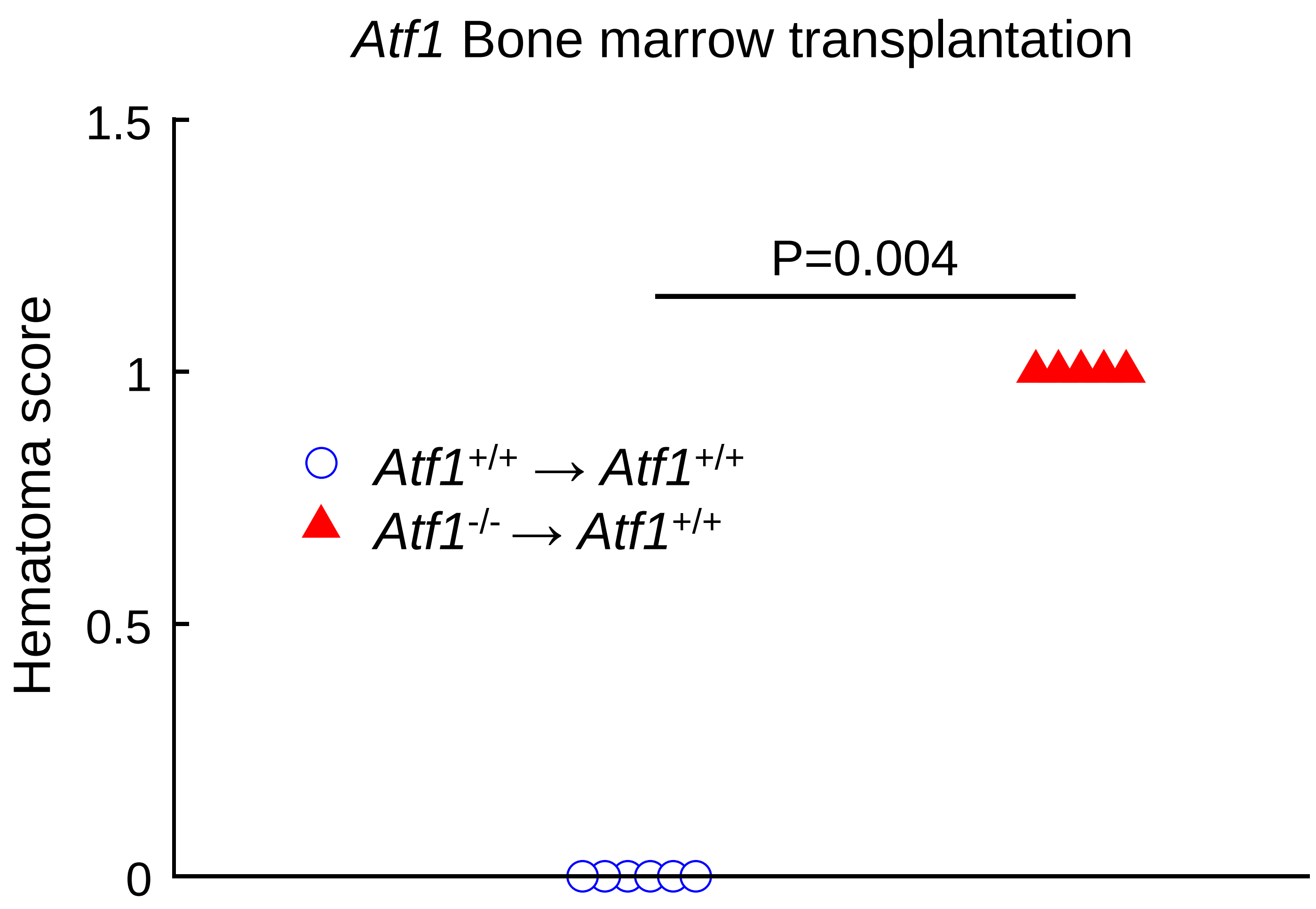

Online Figure II

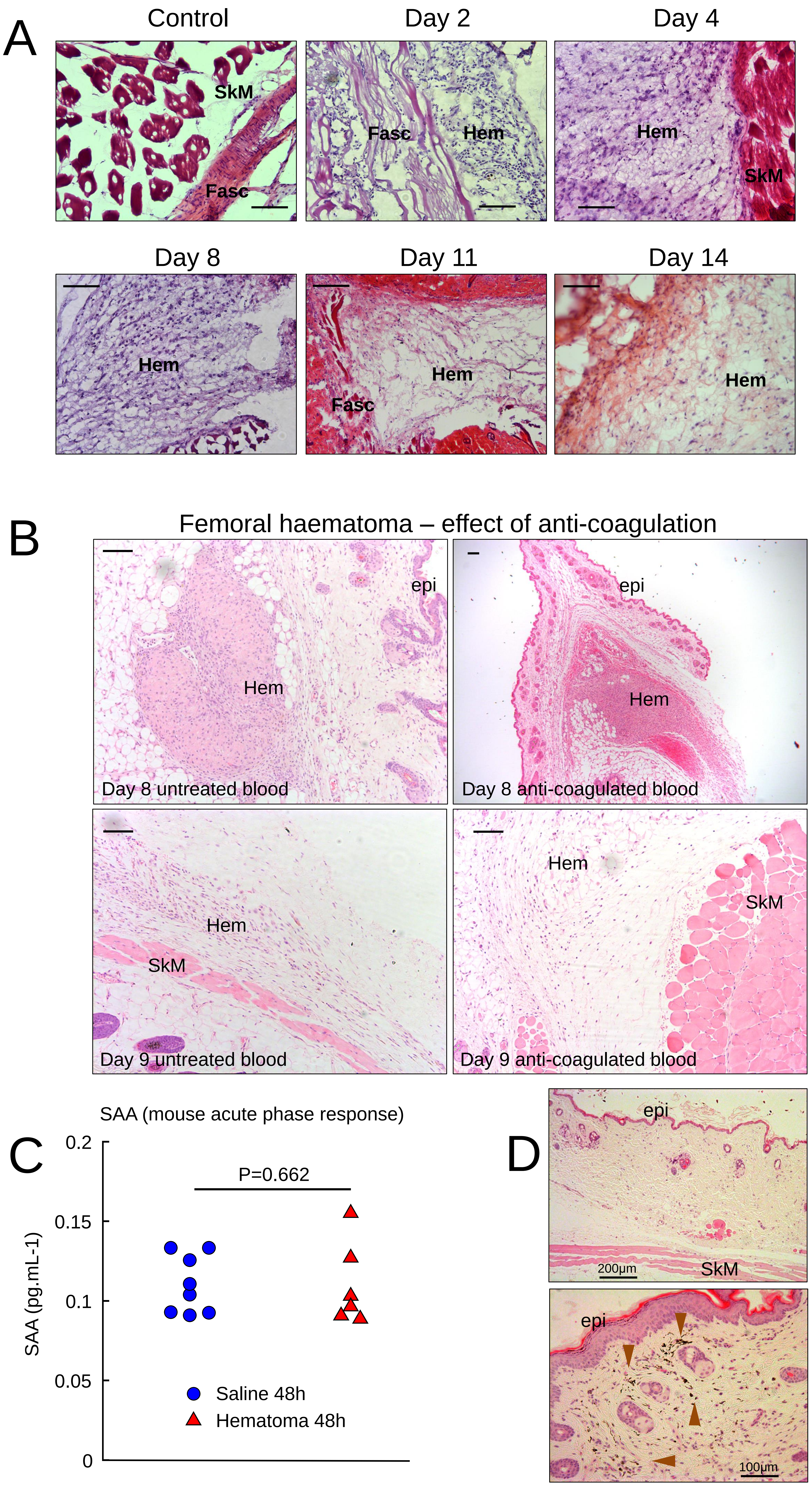

Online Figure III

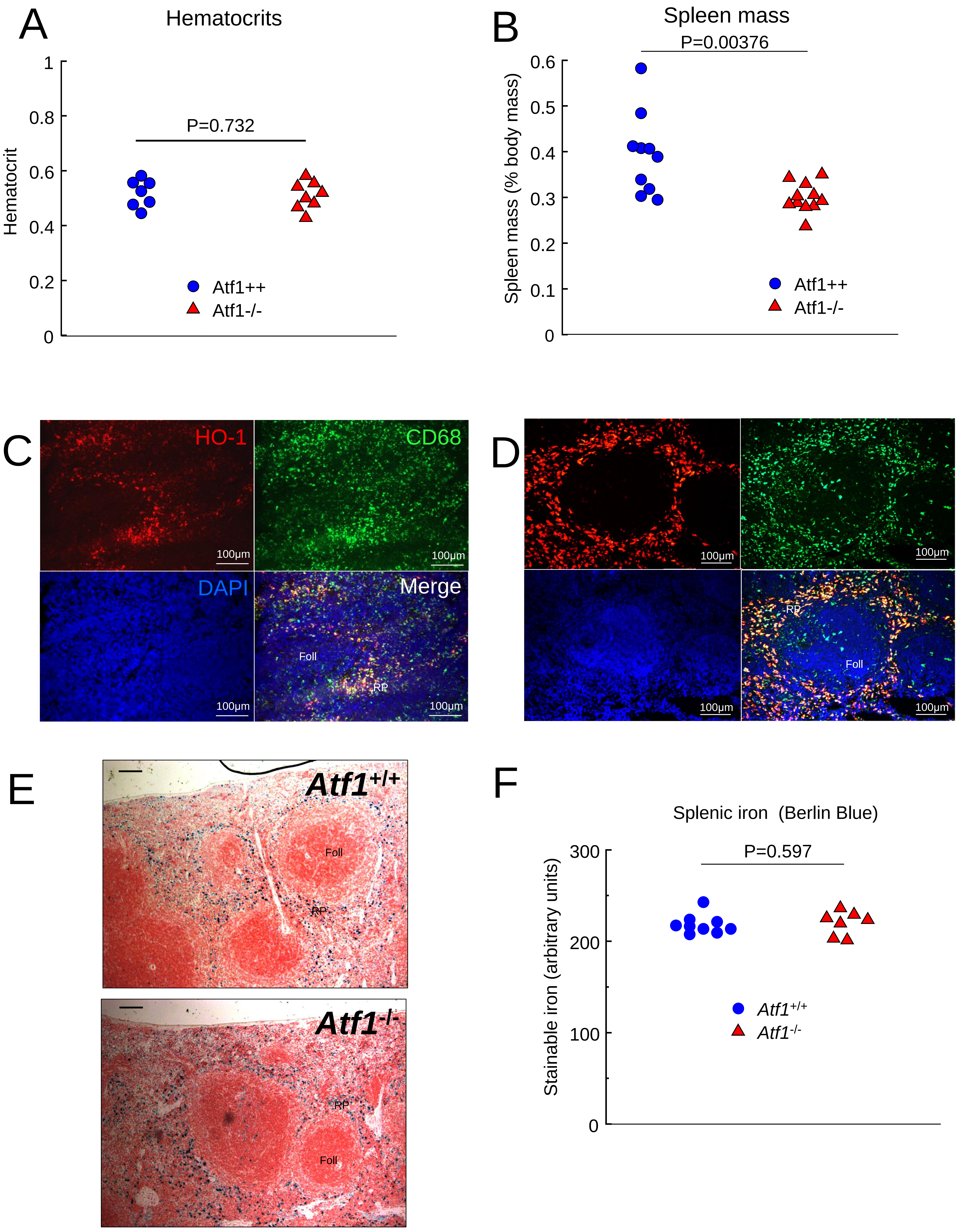

Online Figure IV

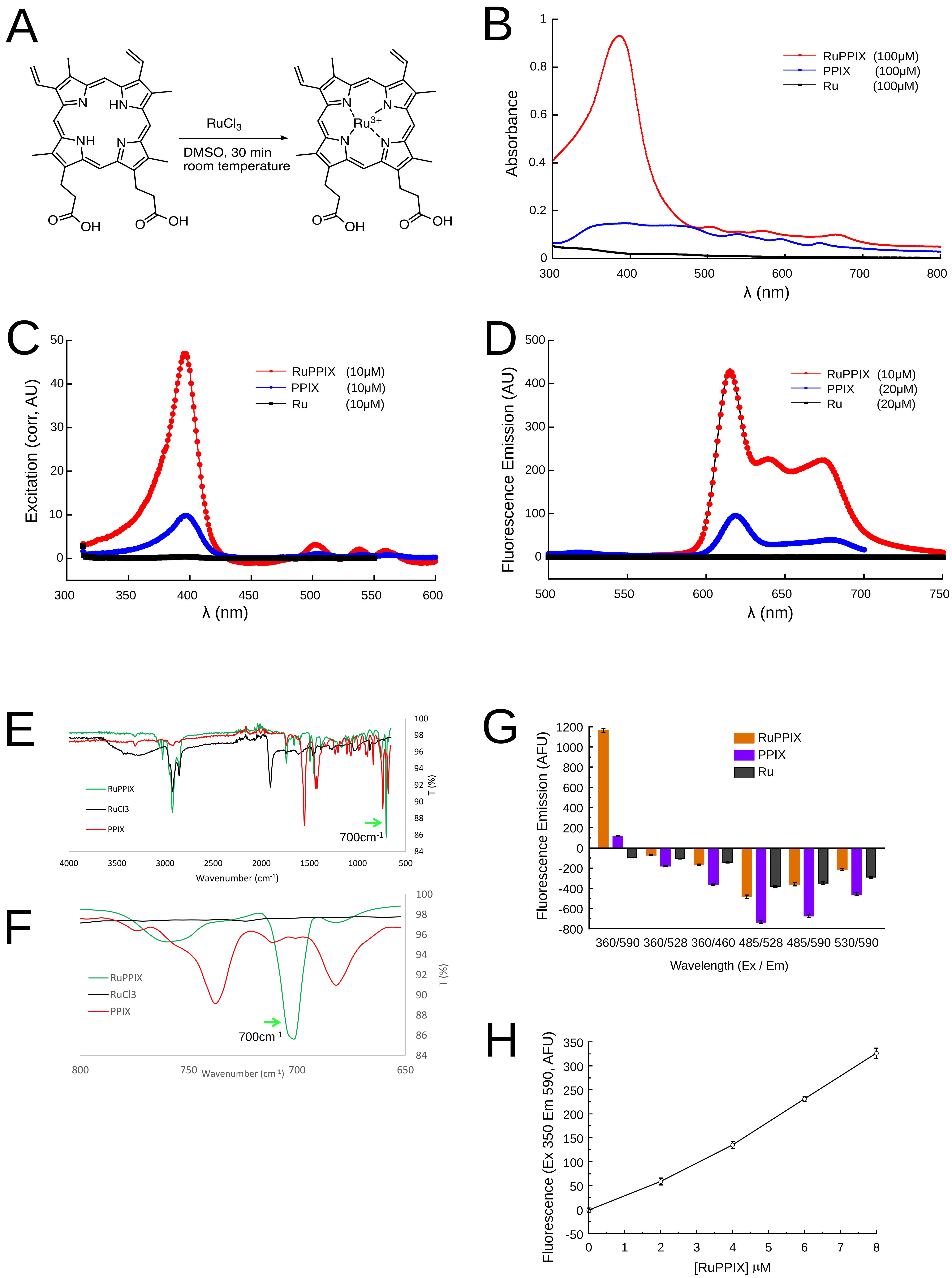

Online Figure V

Validation of Ru^{3+} as fluorescent tracer for Fe – Ru-associated cell fluorescence

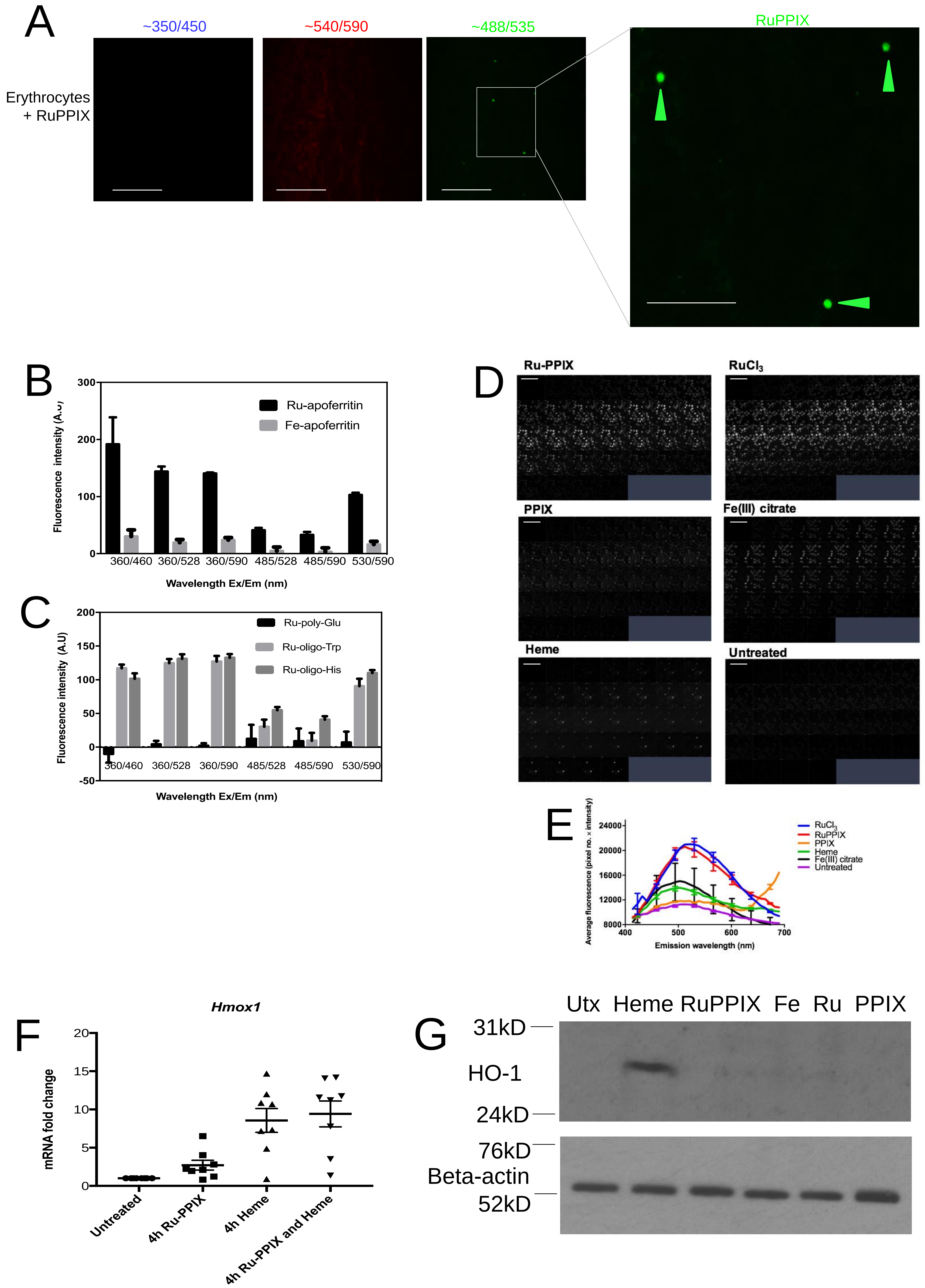

Online Figure VI

A Small macrophages in experimental hematomas express HO-1

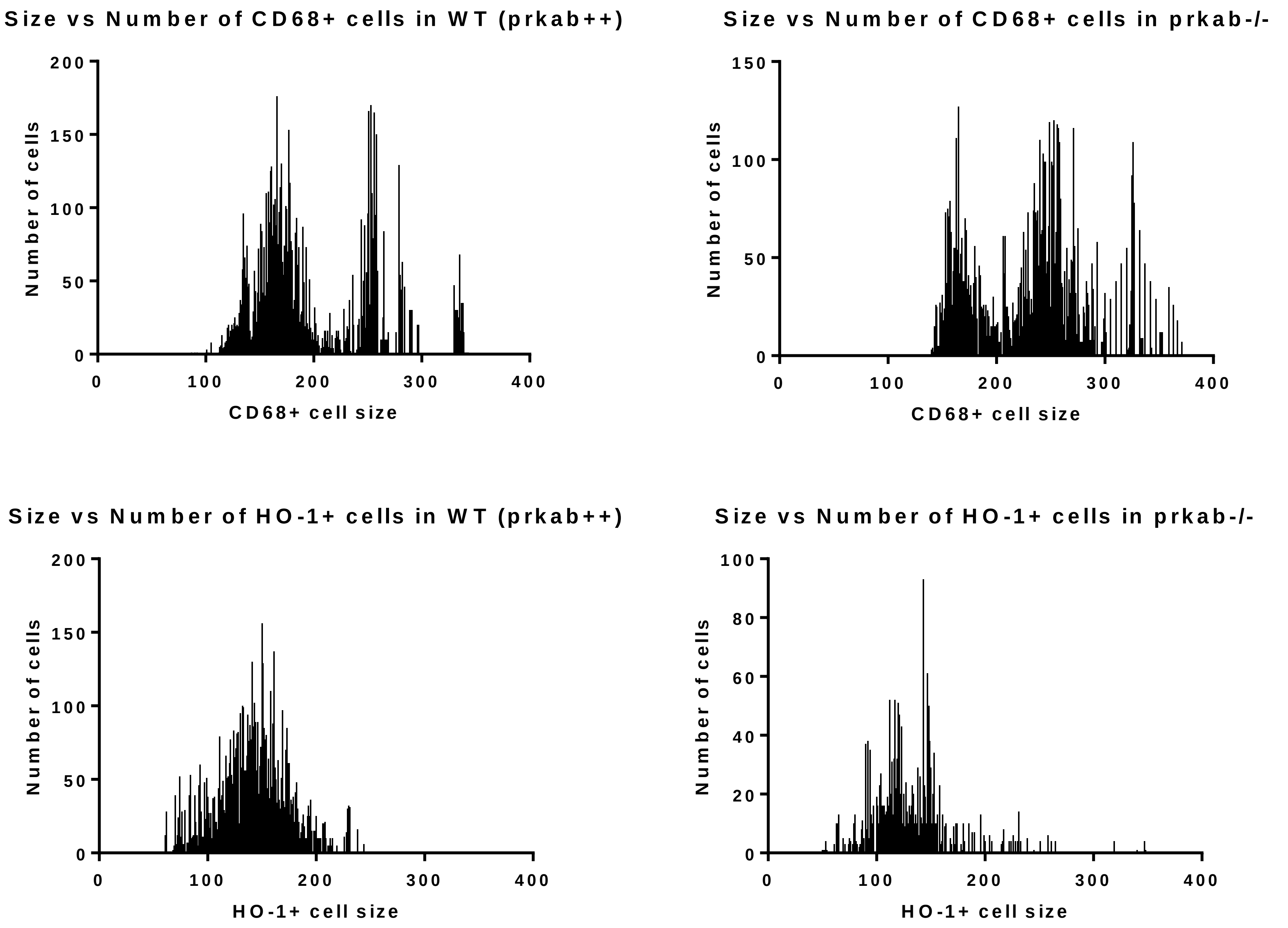

B Ru tracks into small macrophages in experimental hematomas

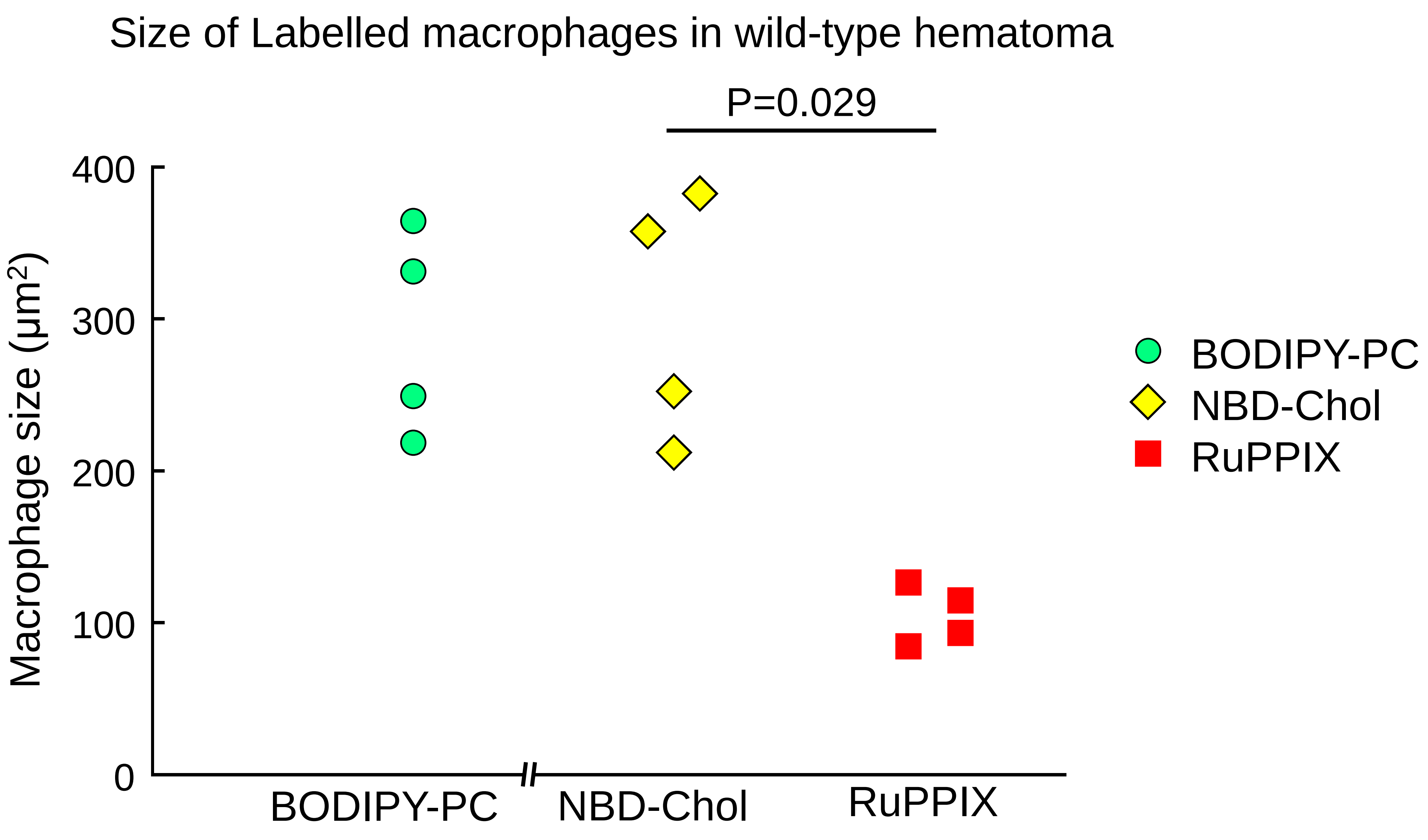

Online Figure VII

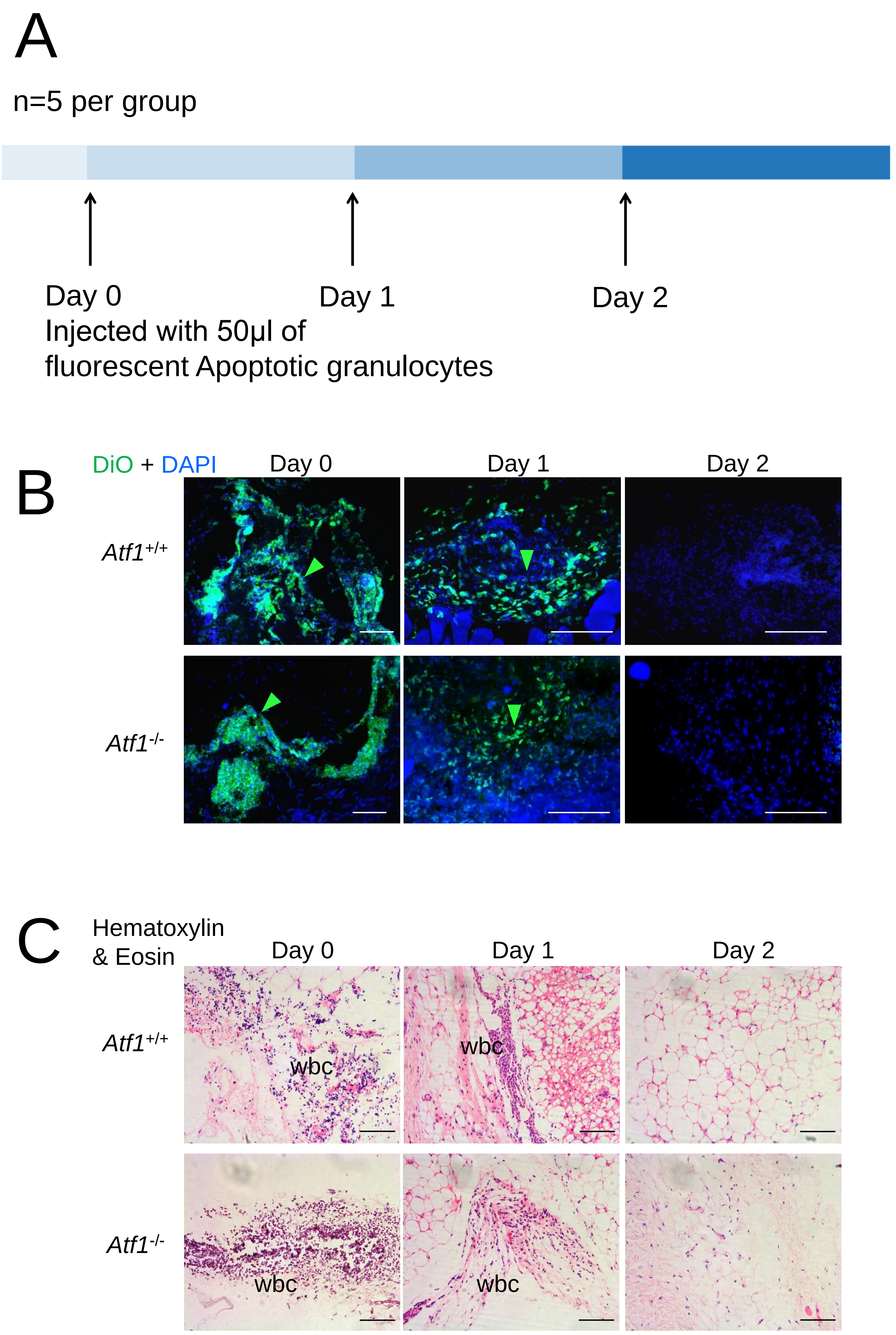

Major Resources Table

In order to allow validation and replication of experiments, all essential research materials listed in the Methods should be included in the Major Resources Table below. Authors are encouraged to use public repositories for protocols, data, code, and other materials and provide persistent identifiers and/or links to repositories when available. Authors may add or delete rows as needed.

Animals (in vivo studies)

Species	Vendor or Source	Background Strain	Sex	Persistent ID / URL
Mouse	Charles River	C57BL/6	M+F	

Genetically Modified Animals

	Species	Vendor or Source	Background Strain	Other Information	Persistent ID / URL
**Parent - Male**	Mouse	KOMP	C57BL/6	Prkab1-/-	Prkab1$^{tm1a(KOMP)Wtsi}$ https://www.komp.org/geneinfo.php?geneid=75288
**Parent - Female**	Mouse	KOMP	C57BL/6	Prkab1-/-	Prkab1$^{tm1a(KOMP)Wtsi}$ https://www.komp.org/geneinfo.php?geneid=75288
**Parent - Male**	Mouse	EMMA	C57BL/6	Atf1-/-	B6.129-Atf1^{tm1Gsc}/Ieg https://www.infrafrontier.eu/search?keyword=atf1
**Parent - Female**	Mouse	EMMA	C57BL/6	Atf1-/-	B6.129-Atf1^{tm1Gsc}/Ieg https://www.infrafrontier.eu/search?keyword=atf1
**Parent - Male**	Mouse	Jax	C57BL/6	LysmCre	B6.129P2-*Lyz2*$^{tm1(cre)Ifo}$/J https://www.jax.org/strain/004781
**Parent - Female**	Mouse	Jax	C57BL/6	LysmCre	B6.129P2-*Lyz2*$^{tm1(cre)Ifo}$/J https://www.jax.org/strain/004781
**Parent - Male**	Mouse	In-house	C57BL/6	Prkab1-/-	Prkab1$^{tm1a(KOMP)Wtsi}$ https://www.komp.org/geneinfo.php?geneid=75288
**Parent - Female**	Mouse	In-house	C57BL/6	Flp	B6.129S4-*Gt(ROSA)26Sor*$^{tm1(FLP1)Dym/}$RainJ https://www.jax.org/strain/009086
**Parent - Male**	Mouse	In-house	C57BL/6	Flp	B6.129S4-*Gt(ROSA)26Sor*$^{tm1(FLP1)Dym/}$Rain https://www.jax.org/strain/009086 J
**Parent - Female**	Mouse	In-house	C57BL/6	Prkab1-/-	Prkab1$^{tm1a(KOMP)Wtsi}$ https://www.komp.org/geneinfo.php?geneid=75288
**Parent - Male**	Mouse	In-house	C57BL/6	Prkab1Fl/Fl	From Flp/Prkab1-/- matings
**Parent - Female**	Mouse	In-house	C57BL/6	LysmCre	B6.129P2-*Lyz2*$^{tm1(cre)Ifo}$/J https://www.jax.org/strain/004781
**Parent - Male**	Mouse	In-house	C57BL/6	LysmCre	B6.129P2-*Lyz2*$^{tm1(cre)Ifo}$/J https://www.jax.org/strain/004781
**Parent - Female**	Mouse	In-house	C57BL/6	Prkab1Fl/Fl	From Flp/Prkab1-/- matings

DOI [to be added]

Antibodies

Target antigen	Vendor or Source	Catalog #	Working concentration	Lot # (preferred but not required)	Persistent ID / URL
Spectrin (SPTB)	Abcam	ab11751	10µg/mL		https://www.abcam.com/alpha-1-spectrin-antibody-17c7-ab11751.html
IgG1kappa Isotype Control	Abcam	ab170190	10µg/mL		https://www.abcam.com/mouse-igg1-kappa-monoclonal-15-6e10a7-isotype-control-ab170190.html
ATF1 (phospho S63)	Abcam	ab76085	4µg/mL		https://www.abcam.com/atf1-phospho-s63-antibody-ep15912y-ab76085.html
Control rabbit monoclonal IgG	Abcam	ab172730	5µg/mL		https://www.abcam.com/rabbit-igg-monoclonal-epr25a-isotype-control-ab172730.html
HO-1 (HMOX1)	Abcam	ab13243	5µg/mL		https://www.abcam.com/heme-oxygenase-1-antibody-ab13243.html
CD68	Serotec (Biorad)	MCA1957	5µg/mL		https://www.bio-rad-antibodies.com/monoclonal/mouse-cd68-antibody-fa-11-mca1957.html?f=purified
Rat IgG2a isotype negative control	Serotec (Biorad)	MCA1212	5µg/mL		https://www.bio-rad-antibodies.com/control/rat-igg2a-negative-control-mca1212.html?f=purified
NF-kB p65 (RELA)	Abcam	Ab32536	2µg/mL		https://www.abcam.com/nf-kb-p65-antibody-e379-ab32536.html
iNOS (NOS2)	Abcam	Ab15323	4µg/mL		https://www.abcam.com/inos-antibody-ab15323.html
3-nitro-Tyrosine	Abcam	Ab110282	5µg/mL		https://www.abcam.com/3-nitrotyrosine-antibody-7a12af6-ab110282.html
Mouse IgG2b, kappa monoclonal isotype control	Abcam	Ab170192	5µg/mL		https://www.abcam.com/mouse-igg2b-kappa-monoclonal-7e10g10-isotype-control-ab170192.html
8-oxo-dG	Trevigen	4354-MC-050	5µg/mL		https://trevigen.com/products-services/cell-stress-and-dna-damage/oxidative-damage/8-oxo-dg-elisa-kit-antibodies/anti-8-oxo-dg-monoclonal-antibody/
Perilipin-2 (PLIN2)	Novus	Nb110-40877	5µg/mL		https://www.novusbio.com/products/perilipin-2-adfp-antibody_nb110-40877
Rabbit IgG	Novus	Nbp2-42891	5µg/mL		https://www.novusbio.com/products/igg-isotype-control_nbp2-24891
Secondary Rabbit Anti-Rat IgG Antibody, mouse adsorbed	Vector	BA-4001	1.25µg/mL		https://vectorlabs.com/biotinylated-rabbit-anti-rat-igg-antibody-mouse-adsorbed.html

DOI [to be added]

(H+L)					
Secondary Goat Anti-Rabbit IgG Antibody (H+L),	Vector	BA-1000	3.75µg/mL		https://vectorlabs.com/biotinylated-goat-anti-rabbit-igg-antibody.html
Alexa Fluor 488 Tyramide SuperBoost Kit, streptavidin	Thermo Fisher	B40932	1:100 dilution		https://www.thermofisher.com/order/catalog/product/B40932?SID=srch-srp-B40932#/B40932?SID=srch-srp-B40932

DNA/cDNA Clones

Clone Name	Sequence	Source / Repository	Persistent ID / URL
N/A			

Cultured Cells

Name	Vendor or Source	Sex (F, M, or unknown)	Persistent ID / URL
RAW264.7	ECACC	M	ECACC 85062803
mBMDM	Mouse femur, in-house	M+F	N/A

Data & Code Availability

Description	Source / Repository	Persistent ID / URL
Genespring	Agilent	https://www.agilent.com/en/products/software-informatics/life-sciences-informatics/genespring-gx
Panther GO	Thomas Lab at University of Southern California	http://www.pantherdb.org/
Venn	Chris Seidel	http://www.pangloss.com/seidel/Protocols/venn.cgi
X2K	Mount Sinai School of Medicine (Avi Ma’ayan)	http://www.maayanlab.net/X2K/
PASTAA	Max Planck Institut	http://trap.molgen.mpg.de/PASTAA.htm
DAVID-GO	Frederick National Laboratory for Cancer Research	https://david.ncifcrf.gov/
LASAGNA 2.0	University of Connecticut	https://biogrid-lasagna.engr.uconn.edu/lasagna_search/index.php

Other

Description	Source / Repository	Persistent ID / URL

DOI [to be added]

Primary Antibodies

Anti-alpha 1 Spectrin antibody [17C7] (ab11751)
https://www.abcam.com/alpha-1-spectrin-antibody-17c7-ab11751.html

Mouse IgG1, kappa monoclonal [15-6E10A7] - Isotype Control (ab170190)
https://www.abcam.com/mouse-igg1-kappa-monoclonal-15-6e10a7-isotype-control-ab170190.html

Anti-ATF1 (phospho S63) antibody [EP1591(2)Y] (ab76085)
https://www.abcam.com/atf1-phospho-s63-antibody-ep15912y-ab76085.html

Recombinant Rabbit IgG, monoclonal [EPR25A] - Isotype Control (ab172730)
https://www.abcam.com/rabbit-igg-monoclonal-epr25a-isotype-control-ab172730.html

Anti-Heme Oxygenase 1 antibody (ab13243) (Abcam Rabbit IgG isotype control as above)
https://www.abcam.com/heme-oxygenase-1-antibody-ab13243.html

CD68 antibody | FA-11
https://www.bio-rad-antibodies.com/monoclonal/mouse-cd68-antibody-fa-11-mca1957.html?f=purified

Rat IgG2a Negative Control antibody
https://www.bio-rad-antibodies.com/control/rat-igg2a-negative-control-mca1212.html?f=purified

Recombinant Anti-NF-kB p65 antibody [E379] (ab32536) (Abcam Rabbit IgG isotype control as above)
https://www.abcam.com/nf-kb-p65-antibody-e379-ab32536.html

Anti-iNOS antibody (ab15323) (Rabbit IgG isotype control as above)
https://www.abcam.com/inos-antibody-ab15323.html

Anti-3-Nitrotyrosine antibody [7A12AF6] (ab110282)
https://www.abcam.com/3-nitrotyrosine-antibody-7a12af6-ab110282.html

Mouse IgG2b, kappa monoclonal [7E10G10] - Isotype Control (ab170192)
https://www.abcam.com/mouse-igg2b-kappa-monoclonal-7e10g10-isotype-control-ab170192.html

Anti-8-oxo-dG Monoclonal Antibody (Mouse IgG2b isotype control as above)
https://trevigen.com/products-services/cell-stress-and-dna-damage/oxidative-damage/8-oxo-dg-elisa-kit-antibodies/anti-8-oxo-dg-monoclonal-antibody/

Perilipin-2/ADFP Antibody
https://www.novusbio.com/products/perilipin-2-adfp-antibody_nb110-40877

Rabbit IgG Isotype Control
https://www.novusbio.com/products/igg-isotype-control_nbp2-24891

Secondary Antibodies

Rabbit Anti-Rat IgG Antibody, mouse adsorbed (H+L), Biotinylated (BA-4001)

https://vectorlabs.com/biotinylated-rabbit-anti-rat-igg-antibody-mouse-adsorbed.html

Goat Anti-Rabbit IgG Antibody (H+L), Biotinylated (BA-1000)
https://vectorlabs.com/biotinylated-goat-anti-rabbit-igg-antibody.html

Goat Anti-Mouse IgG Antibody (H+L), Biotinylated (BA-9200)
https://vectorlabs.com/biotinylated-goat-anti-mouse-igg-antibody.html

Alexa Fluor 488 Tyramide SuperBoost Kit, streptavidin
https://www.thermofisher.com/order/catalog/product/B40932?SID=srch-srp-B40932#/B40932?SID=srch-srp-B40932

List of Primers

Hprt

F 5- CCT GGT TAA GCA GTA CAG CCC C -3

R 5- CGA CAG GTC CTT TTC ACC AGC A -3

Actab

F 5- AAA CCC GGC GGC GCA AC -3

R 5- GCG CAG CGA TAT CGT CAT CCA -3

Gapdh

F 5- CGG CCA AAT CCG TTC ACA CC -3

R 5- AGA GGG ATG CTG CCC TTA CC -3

Atf1

F 5- GAG TTA CGC TCT CCT CCC -3

R 5- GAG ATG CAG TCT CTG TCG TGT -3

Hmox1

F 5- TGG CGT CAC TTC GTC AGA GG -3

R 5- GGT TTC CCT CGG GGT GTC TC -3

Socs1

F 5- CAG AGA GAA CTG CGG CCG TG -3

R 5-GGC CAA CAG ACC CCA AGG AG -3

Apoe

F 5- ATC CGA TCC CCT GCT CAG AC -3

R 5- AAT CTG ACC AAC AGC ACG GC -3

Nr1h2

F 5- GAC TTC CAC CGT GCA GGC TT -3

R 5- CTT GTG GCT GCC CCT ACT GG -3

Abca1

F 5- GCT CTG CTC CCT GTT TCC CC -3

R 5- TGT GAC TCT GTG GCT GGT CA -3

Nr1h3

F 5- CTT CCT GGA GCC CTG GAC ATT -3

R 5- TCT TGG GTC GCC AGT AGG AAG -3

Igf1

F 5- CGCCACACTGACATGCCCAA -3

R 5- AGGTCTTGTTTCCTGCACTTCCT -3

Spic

F 5- ATC CTC ACG TCA CAG GCA ACG -3

R 5- CGG ATT GGT GGA AGC CTC CTT -3

Heme

**HO-1**	Wild-type (Prkab1++)			Prkab1-/-		
Time point	Mean	SEM	n	Mean	SEM	n
0	1.0260981	0.0120209	6	1.0985118	0.0495308	6
0.5	1.4873545	0.0174634	6	1.245864	0.0245634	6
1	1.9797284	0.4559598	6	1.5498205	0.5691604	6
2	3.2305927	0.307001	6	3.4570653	1.2311875	6
4	15.998713	3.7837958	6	7.9113985	1.4990594	6
8	23.60661	2.2620403	6	6.3736156	0.5093666	6
24	5.9469286	0.857405	6	4.9861793	0.9085376	6
48	4.8914631	0.7354941	6	4.9295409	0.9413896	6
96	3.8737626	0.7371278	6	6.6932475	0.5387638	6

**Atf-1**	Wild-type (Prkab1++)			Prkab1-/-		
Time point	Mean	SEM	n	Mean	SEM	n
0	1.0094085	0.0043494	6	1.0120738	0.004989	6
0.5	1.245864	0.015643	6	0.9645341	0.0014364	6
1	1.4758503	0.2409817	6	0.991966	0.2481524	6
2	2.5571413	0.7711905	6	0.7932278	0.2503384	6
4	2.1536152	0.3497556	6	1.3883875	0.2760127	6
8	2.3357784	0.2328581	6	1.229627	0.2796535	6
24	0.9636865	0.0979917	6	0.9932355	0.213636	6
48	1.146264	0.2766338	6	0.9351797	0.1407682	6
96	1.2609145	0.3916185	6	0.8387784	0.2294486	6

**Socs1**	Wild-type (Prkab1++)			Prkab1-/-		
Time point	Mean	SEM	n	Mean	SEM	n
0	1.0176973	0.0069733	6	1.0279513	0.010011	6
0.5	1.245644	0.004134	6	0.963453	0.002143	6
1	1.6427623	0.3056909	6	0.9982238	0.272602	6
2	2.3880074	0.3474225	6	0.9747361	0.2330738	6
4	1.2413868	0.1253016	6	0.738137	0.1508778	6
8	3.0398079	0.3778015	6	0.9973723	0.1930323	6
24	1.126555	0.2028151	6	0.9627141	0.1187891	6
48	2.3714448	0.4716412	6	1.0808279	0.1075199	6
96	1.4424308	0.2873929	6	0.9418813	0.1640346	6

**LXR-β**	Wild-type (Prkab1++)			Prkab1-/-		
Time point	Mean	SEM	n	Mean	SEM	n
0	1.0211091	0.0159604	6	1.0254498	0.0130589	6
0.5	1.1564753	0.0234534	6	1.0046314	0.0015341	6
1	1.30245	0.1970047	6	1.0084181	0.116252	6
2	1.5469833	0.1988457	6	0.8598211	0.0863485	6
4	1.1261986	0.1137513	6	0.7801636	0.1080602	6
8	0.9840235	0.0444913	6	0.9194021	0.0860553	6
24	0.8417579	0.1517979	6	0.942209	0.1492586	6
48	1.293511	0.3162882	6	1.1330198	0.1751933	6

96	0.9570048	0.1084048	6	0.9345569	0.084941	6

**LXR-α**	Wild-type (Prkab1++)			Prkab1-/-		
Time point	Mean	SEM	n	Mean	SEM	n
0	1.0088164	0.0050924	6	1.0229951	0.0108694	6
0.5	0.9	0.0408248	6	0.7426398	0.0993396	6
1	0.900512	0.0513458	6	0.6692491	0.081622	6
2	0.8783883	0.0703541	6	0.7694349	0.0543424	6
4	0.7826939	0.0657414	6	0.652052	0.0535732	6
8	0.7259646	0.0542132	6	0.743908	0.070287	6
24	0.643756	0.0561898	6	0.892306	0.1306179	6
48	0.749014	0.1294937	6	1.1115728	0.1324845	6
96	1.077569	0.040925	6	0.7574947	0.1249825	6

**ApoE**	Wild-type (Prkab1++)			Prkab1-/-		
Time point	Mean	SEM	n	Mean	SEM	n
0	1.045232	0.01728	6	1.0347609	0.0200279	6
0.5	1.4564864	0.0053435	6	0.7463413	0.0045633	6
1	1.7258351	0.2472963	6	0.4569823	0.0840596	6
2	2.6506939	0.388684	6	0.425857	0.0529373	6
4	1.411017	0.1321213	6	0.492092	0.1130156	6
8	4.0389408	0.7298417	6	0.8108685	0.1812228	6
24	0.7547845	0.0801365	6	1.0387603	0.1271341	6
48	0.874416	0.4045936	6	0.9468041	0.0781084	6
96	2.2267323	0.2600935	6	1.3236542	0.2074206	6

**Abca1**	Wild-type (Prkab1++)			Prkab1-/-		
Time point	Mean	SEM	n	Mean	SEM	n
0	1.0197121	0.0075997	6	1.0088764	0.0045769	6
0.5	1.18	0.0374166	6	0.9694828	0.1152073	6
1	1.5237133	0.1894056	6	0.8106709	0.0822296	6
2	1.6065486	0.2322678	6	0.9973819	0.1444139	6
4	1.1797996	0.1374209	6	0.9850653	0.1753039	6
8	1.3165608	0.1900045	6	0.8871508	0.1458887	6
24	1.0519465	0.1054755	6	0.7129944	0.068003	6
48	1.0508109	0.1622754	6	1.0348111	0.0696997	6
96	1.8044095	0.144123	6	1.094153	0.1826191	6

**Igf1**	Wild-type (Prkab1++)			Prkab1-/-		
Time point	Mean	SEM	n	Mean	SEM	n
0	1.0033346	0.001005	6	1.0193944	0.0141904	6
0.5	1	0.0258199	6	0.942601	0.0855077	6
1	1.0889146	0.0544315	6	0.5483433	0.0961616	6
2	1.1080235	0.0794099	6	0.6432054	0.0760438	6
4	1.1254101	0.0816166	6	0.5456885	0.0622192	6
8	1.2217203	0.1188354	6	0.6366704	0.0660942	6

24	1.5019503	0.3765122	6	0.9773698	0.0774339	6
48	1.298356	0.3975979	6	0.9757795	0.0545169	6
96	1.4465128	0.1034395	6	0.9681733	0.0936616	6

**Pdgf**	Wild-type (Prkab1++)			Prkab1-/-		
Time point	Mean	SEM	n	Mean	SEM	n
0	1.0544699	0.0467671	6	1.033997	0.0092759	6
0.5	1.14	0.0509902	6	0.6763624	0.0707836	6
1	1.2136168	0.1849176	6	0.8610413	0.0745352	6
2	1.0567625	0.14616	6	0.8318775	0.0978057	6
4	1.0634578	0.2384696	6	0.87329	0.0754316	6
8	1.0726419	0.0742359	6	0.9480166	0.075351	6
24	0.8830746	0.1138599	6	0.8806048	0.1833666	6
48	1.2397733	0.144633	6	1.0742348	0.1113795	6
96	1.5063626	0.1101842	6	0.952829	0.1286059	6

HO-1

Time point	Wild-type (Atf1++) Mean	SEM	n	Atf1-/- Mean	SEM	n
0	1.0263332	0.0133351	6	1	0	6
0.5	1.545643	0.015864	6	1.4366588	0.2561421	6
1	1.9357433	0.3042684	6	1.6148247	0.3832144	6
2	3.1932027	0.3521797	6	3.7277102	1.182879	6
4	17.917852	2.2499	6	7.8869862	1.4090264	6
8	29.846053	1.4960117	6	8.1476992	1.6528465	6
24	4.898555	0.7147311	6	4.5478358	0.5349502	6
48	4.2525324	0.3972358	6	3.901861	0.5070995	6
96	3.2226175	0.5827882	6	2.5702052	0.3656663	6

Socs1

Time point	Wild-type (Atf1++) Mean	SEM	n	Atf1-/- Mean	SEM	n
0	1.046639	0.011521	6	1	0	6
0.5	1.1592208	0.012927	6	0.3948292	0.0758019	6
1	1.5810735	0.1937183	6	0.2536564	0.0769876	6
2	2.34685	0.1426507	6	0.548387	0.125806	6
4	1.233821	0.1260249	6	0.3026492	0.0702504	6
8	3.1627415	0.1152502	6	0.529333	0.1738273	6
24	1.1062902	0.0555402	6	1.4014728	0.3483294	6
48	2.5713492	0.1311497	6	3.2076727	0.8145117	6
96	1.5379308	0.189011	6	1.0439545	0.1300928	6

LXR-β

Time point	Wild-type (Atf1++) Mean	SEM	n	Atf1-/- Mean	SEM	n
0	1.0126862	0.0056126	6	1	0	6
0.5	1.01362	0.0038581	6	0.8717014	0.1438572	6
1	1.191038	0.1284405	6	0.8572358	0.1279767	6
2	1.5453793	0.1337478	6	0.9050158	0.118617	6
4	1.1512658	0.0762064	6	0.6945116	0.1174241	6
8	1.0314592	0.0349015	6	1.0571463	0.1343707	6
24	0.9880147	0.0437901	6	1.0551541	0.0730364	6
48	1.2938563	0.1101209	6	0.8654788	0.113941	6

96	1.0367503	0.0443712	6	0.7709172	0.1843272	6

**LXR-α**	Wild-type (Atf1++)			Atf1-/-		
Time point	Mean	SEM	n	Mean	SEM	n
0	1.0042025	0.0008848	6	1	0	6
0.5	1.027598	0.0166383	6	0.7426398	0.0993396	6
1	0.956199	0.0227721	6	0.7266394	0.1052415	6
2	0.923685	0.0705576	6	0.8101288	0.1055105	6
4	0.9226112	0.0457044	6	0.7298052	0.1192392	6
8	0.8319583	0.030448	6	1.3507908	0.1821097	6
24	0.707205	0.0199871	6	0.9910195	0.0865788	6
48	0.7294548	0.1476532	6	0.8284767	0.1126342	6
96	1.1249538	0.0445247	6	0.6627193	0.1156187	6

**ApoE**	Wild-type (Atf1++)			Atf1-/-		
Time point	Mean	SEM	n	Mean	SEM	n
0	1.0342045	0.0105934	6	1	0	6
0.5	1.4910283	0.0743814	6	1.1052918	0.1351432	6
1	1.9231793	0.3373776	6	0.9568916	0.1066679	6
2	2.9225722	0.3634509	6	1.1921436	0.1795458	6
4	1.3949595	0.1211046	6	0.969314	0.1973047	6
8	4.6761873	0.5618922	6	0.8921224	0.1287518	6
24	0.9046653	0.1077279	6	1.0080078	0.1178081	6
48	1.0628463	0.1300901	6	0.8208997	0.1541507	6
96	2.2042302	0.1607455	6	1.8251238	0.0464409	6

**Abca1**	Wild-type (Atf1++)			Atf1-/-		
Time point	Mean	SEM	n	Mean	SEM	n
0	1.0152637	0.0055042	6	1	0	6
0.5	1.2289556	0.0360715	6	0.9694828	0.1152073	6
1	1.4800717	0.1485102	6	0.7577832	0.0674891	6
2	1.4624697	0.2382704	6	0.9184008	0.1343155	6
4	1.2121313	0.0891623	6	0.9305314	0.074417	6
8	1.2723708	0.1768761	6	0.9348908	0.1165802	6
24	1.1126698	0.1254031	6	0.7948765	0.0624641	6
48	0.9398678	0.1729979	6	0.9964607	0.147988	6
96	1.9538317	0.0487168	6	0.8239912	0.0705034	6

**Igf1**	Wild-type (Atf1++)			Atf1-/-		
Time point	Mean	SEM	n	Mean	SEM	n
0	1.0073183	0.0054915	6	1	0	6
0.5	1.041004	0.0234789	6	0.942601	0.0855077	6
1	1.1248555	0.1010341	6	1.0051448	0.0431761	6
2	1.2013495	0.0791266	6	0.8403032	0.1118774	6
4	1.2161317	0.0945908	6	0.8440504	0.1094845	6
8	1.236711	0.1748958	6	1.0649842	0.1252235	6

24	1.4292553	0.1444762	6	0.9580418	0.0616307	6
48	1.2640737	0.2528839	6	0.9139878	0.0764253	6
96	1.5781272	0.1043017	6	1.0675833	0.0726143	6

**Pdgf**	Wild-type (Atf1++)			Atf1-/-		
Time point	Mean	SEM	n	Mean	SEM	n
0	1.014082	0.0072258	6	1	0	6
0.5	1.2116608	0.0448895	6	0.6763624	0.0707836	6
1	1.3299445	0.1487403	6	0.5591644	0.0952127	6
2	0.993922	0.1156389	6	0.6691368	0.088498	6
4	1.0803415	0.1605975	6	0.7756732	0.2009276	6
8	1.0453883	0.0735665	6	0.863886	0.1045637	6
24	0.8378007	0.099916	6	0.8005447	0.0835748	6
48	1.4317137	0.1412551	6	1.0579018	0.1331085	6
96	1.8501568	0.2158209	6	0.5963333	0.1568277	6

Full unedited gels for Supplemental Figure 5G

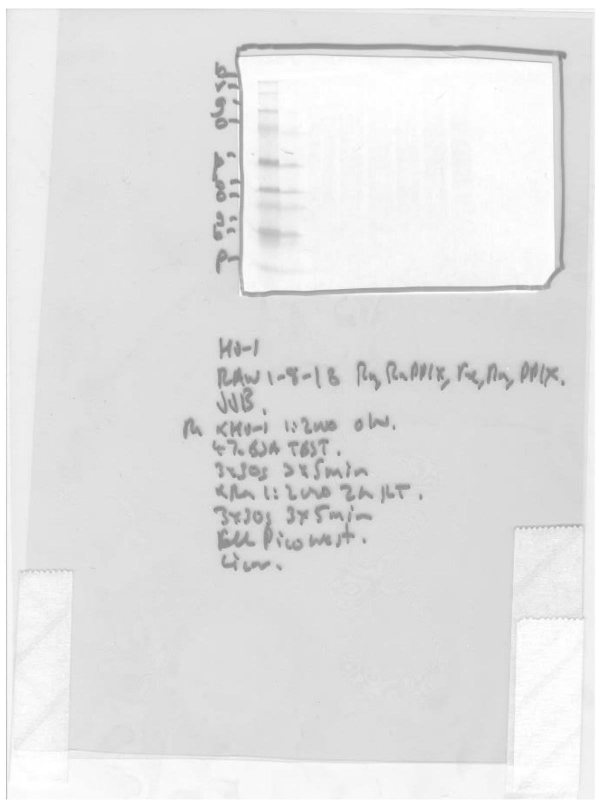

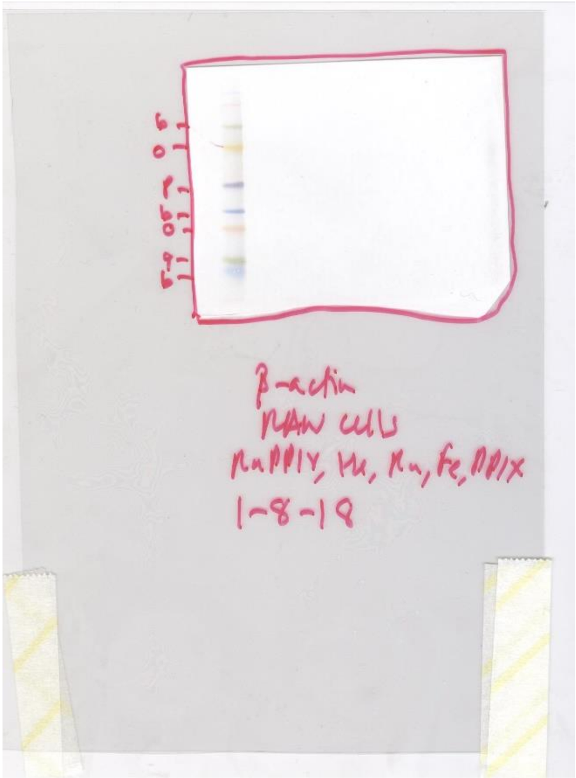

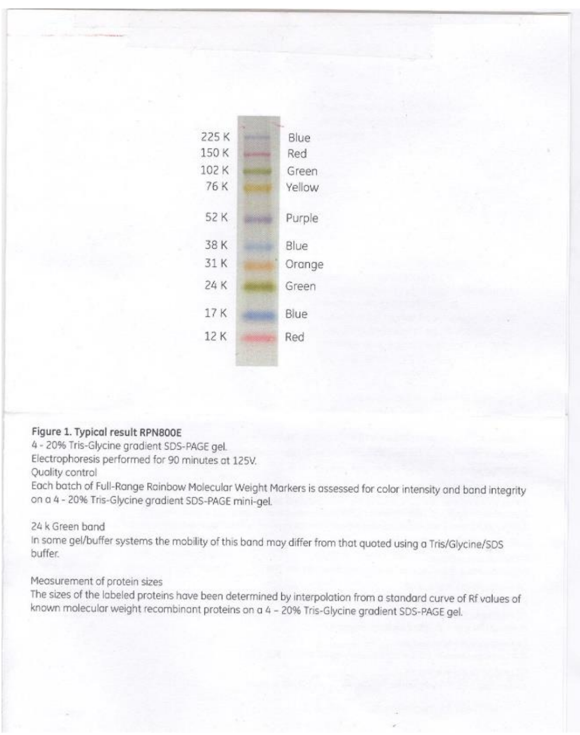

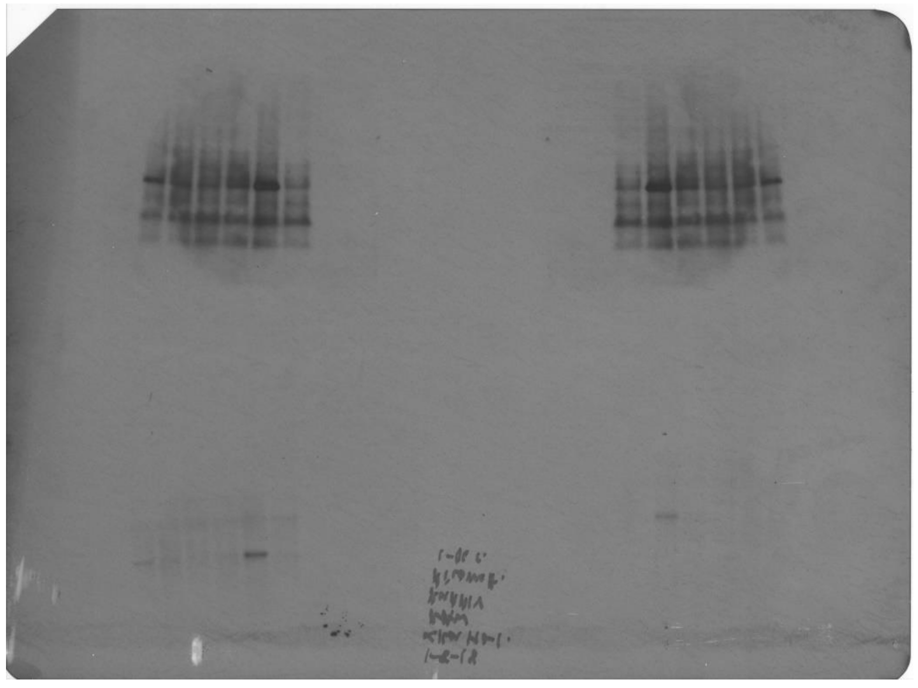

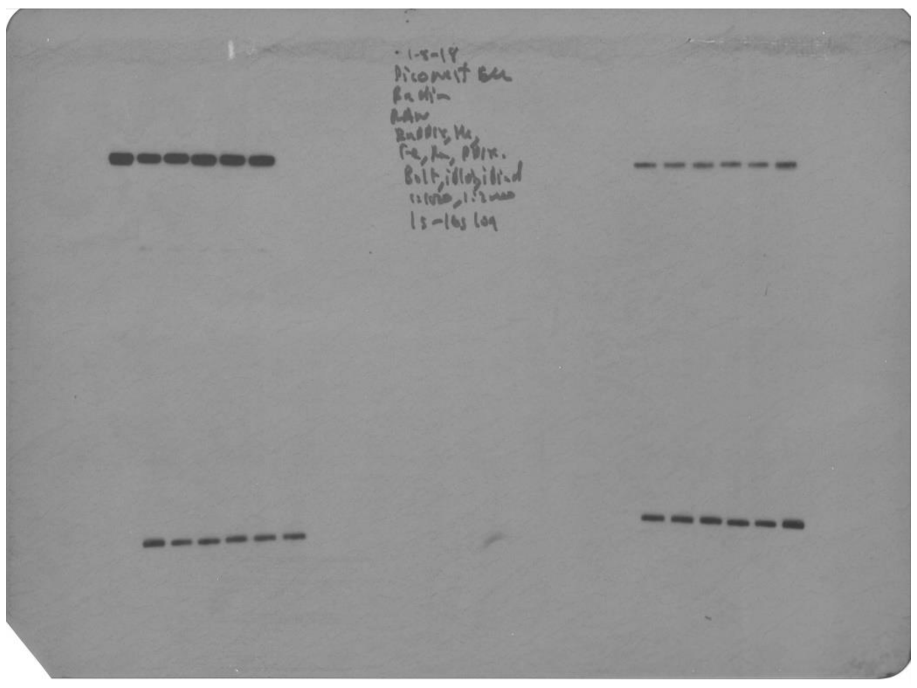

Supplement: Supplementary file 2 [file res-127-928-s002.pdf]
